# Synthesis and cytotoxicity against tumor cells of pincer N-heterocyclic ligands and their transition metal complexes†

**DOI:** 10.1039/d1ra05918a

**Published:** 2021-10-27

**Authors:** Afaf Oulmidi, Smaail Radi, Abderrazak Idir, Abdelmajid Zyad, Imad Kabach, Mohamed Nhiri, Koen Robeyns, Aurelian Rotaru, Yann Garcia

**Affiliations:** LCAE, Department of Chemistry, Faculty of Sciences, University Mohamed I BP 524 60 000 Oujda Morocco s.radi@ump.ac.ma yann.garcia@uclouvain.be +212-10472330; Institute of Condensed Matter and Nanosciences, Molecular Chemistry, Materials and Catalysis (IMCN/MOST), Université catholique de Louvain Belgium; Team of Experimental Oncology and Natural Substances, Cellular and Molecular Immunopharmacology, Faculty of Sciences and Techniques, Sultan Moulay Slimane University Mailbox 523 23000 Beni Mellal Morocco; Laboratory of Biochemistry and Molecular Genetics, Faculty of Sciences and Technology Tangier Morocco; Department of Electrical Engineering and Computer Science, MANSiD Research Center, “Stefan cel Mare” University University Street, 13 Suceava 720229 Romania

## Abstract

The complexes: [CoL_2_](ClO_4_)_2_ (1), [FeL_2_](ClO_4_)_2_ (2), [NiL_2_](ClO_4_)_2_ (3) and [MnLCl_2_] (4), with L = diethyl-1,1′-(pyridine-2,6-diyl)bis(5-methyl-1*H*-pyrazole-3-carboxylate), were synthesized and fully characterized. Structural analysis revealed two distinct patterns influenced by the counter ions where L acts as a tridentate chelating ligand. The *in vitro* antitumor activity of L and L′ (diethyl 2,2′-(pyridine-2,6-diylbis(5-methyl-1*H*-pyrazole-3,1-diyl)) diacetate) as well as their metal complexes, was tested by the measurement of their cytostatic and cytotoxic properties towards the blood cancer mastocytoma cell line P815. We have also investigated their interactions with the antioxidant enzyme system. As a result, [MnL′Cl_2_] (1′) exhibited the strongest activity compared to reference cis-platin with no cytotoxicity towards normal cells PBMCs (Peripheral Blood Mononuclear Cells). On the other hand, the antioxidant enzyme activity showed that the efficiency of metal complex 1′ against P815 tumor cells was *via* the rise in the SOD activity and inhibition of CAT enzyme activity. This proof of concept study allows disclosure of a new class of molecules in cancer therapeutics.

## Abstract 2

The complexes: [CoL_2_](ClO_4_)_2_ (1), [FeL_2_](ClO_4_)_2_ (2), [NiL_2_](ClO_4_)_2_ (3) and [MnLCl_2_] (4), with L = diethyl-1,1′-(pyridine-2,6-diyl)bis(5-methyl-1*H*-pyrazole-3-carboxylate), were synthesized and fully characterized.

## Graphical abstract

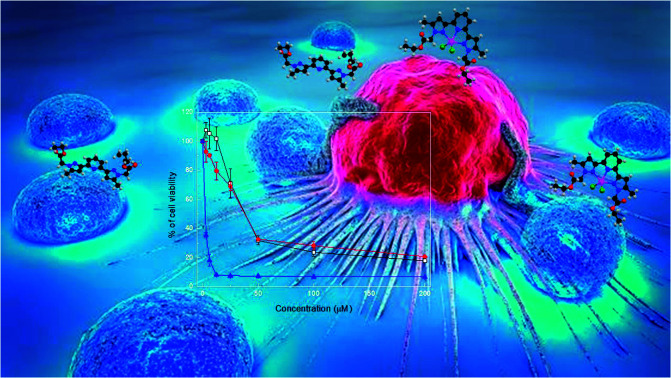

## 1. Introduction

According to the World Health Organization (WHO), cancer remains one of the world's most damaging diseases, with 18.1 million new cases of cancer and not less than 9.6 million deaths in 2018.^1^ This is far more than the Covid-19 pandemic, which currently threatens our daily life and economic growth. The situation is particularly worrying in Europe, with 23% of reported cancers and 20% of deaths while in the United States, it is considered as the second leading cause of death, after heart disease.^2^ Therefore, developing an urgent effective treatment remains one of the greatest challenges in clinical oncology. Treatment options for cancer depend on the type of islet cell cancer, the extent of metastasis, and the general health of a patient. Standard treatment options for cancer include surgery, chemotherapy, hormone therapy, radiotherapy or the introduction of biological drugs. The emergence of resistant tumours, however, considerably limits the effectiveness of conventional chemotherapies. This is why it is becoming interesting to explore various therapeutic avenues, including the development of new drugs that are active on resistant cancers and prevent the formation of metastases. Heterocycles represent a majority of active components of cancer agents,^3^ some of them being coordinated to metal complexes. For example, nitrogen-donor chelating ligands have been widely used in the architectural design of metal complexes with biological applications, including cytotoxic activities.^4–7^ For instance, Singh *et al.* synthesized a series of 5-iodouracil complexes with Mn(ii), Co(ii), Cu(ii), Zn(ii) and Cd(ii) ions, some of which were active against Sarcoma-180 and L929 tumor cells.^8^ Later, the effects of 5-bromouracil complexes with Cr(iii), Fe(iii) and Al(iii) were reported on P815 murine mastocytoma.^9^ We recall that P815 is a mastocytoma cell line commonly used as an experimental tumor model due to its several advantages for *in vivo* experimentation. It is derived by methylcholanthrene treatment of a male DBA/2 mouse.^10^ Other examples include transition metal complexes used as pro-drugs where a cytotoxic agent bound to the metal ion can be released during treatment.^11^

However, such complexes are limited by severe side effects, general toxicity, and drug resistance. To limit these side effects, manganese is among the most promising metals that is essential to human physiology. In the human body, manganese is one of the components required for Mn superoxide dismutase which is primarily responsible for trapping reactive oxygen species in mitochondrial oxidative stress.^12^ It is also involved in the synthesis and activation of numerous enzymes and in the regulation of glucose and lipid metabolism in humans.^13^ In addition to its physiological role, manganese may have beneficial therapeutic and preventive effects on infectious diseases and, compared to other metal-based drugs, Mn(ii) complexes generally exert lower toxicity and have less side effects.

Heterocyclic compounds containing pyrazole and pyridine were reported to show remarkable biological properties.^14,15^ The pyrazole scaffold is a privileged pharmacophore encountered in many chemical compounds confirmed to be associated with various biological assets such as anti-inflammatory,^16^ antimicrobial and anticancer properties.^17,18^ Furthermore, many compounds bearing a pyridine moiety have been identified to be biologically active^19–22^ and therapeutically relevant in medicinal chemistry and to display versatile biological activities including antibacterial and anticancer activities.^18,23^ In view of their biological significant activity, the combination of pyrazole and pyridine moieties may lead to the synthesis of a new class of therapeutical active compounds, and is thus very much attractive.^24,25^ In particular, because such materials are rarely cited for their individual anticancer activities, despite their significant activities,^26,27^ which could be attributed to the electronic interactions between the metal centre and π electrons in their aromatic rings.^28,29^

**Scheme 1:**
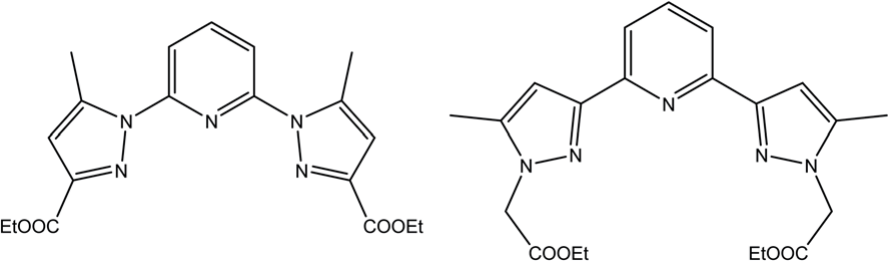
Ligand L (left) and L′ (right) respectively.

To this end, we have designed novel derivatives of nitrogen-chelating pyrazolyl–pyridine ligands with CC (L′) and CN (L) junctions, to study their *in vitro* individual anticancer activities with particular emphasis on their complexes of Co, Fe, Ni, and Mn. This led us to prepare diethyl 2,2′-(pyridine-2,6-diylbis(5-methyl-1*H*-pyrazole-3,1-diyl)) diacetate (L′)^30^ and diethyl 1,1′-(pyridine-2,6-diyl) bis(5-methyl-1*H*-pyrazole-3-carboxylate) (L) (Scheme 1). Metal complexes [CoL_2_](ClO_4_)_2_ (1), [FeL_2_](ClO_4_)_2_ (2), [NiL_2_](ClO_4_)_2_ (3), [MnLCl_2_] (4), [MnL′Cl_2_] (1′) and [CdL′Cl_2_] (2′) were also investigated. A structure–activity relationship study taking into account the nature of ligands and coordination metals is discussed.

## 2. Experimental section

### 2.1. Materials and methods

All solvents and chemicals were obtained from commercial sources and used without purification. The progress of reactions and the elution of products were followed by TLC (silica gel). Infrared spectra were recorded on a PerkinElmer 1310 Spectrometer FT-IR in the region 4000–400 cm^−1^. ^1^H and ^13^C NMR spectra were acquired on a Bruker AC 300 MHz instrument. Chemical shifts (*δ*) are given in ppm referring to the signal centre using the solvent peaks as reference: CDCl_3_ 7.26 ppm/77.36 ppm. High-resolution mass spectrometry HRMS data were obtained with a Q Exactive Thermofisher Scientific ion trap spectrometer using ESI ionization. UV-visible spectra were recorder using a Shimadzu 3600 plus spectrometer equipped with Harrick praying mantis modulus which allows direct analysis of powders in reflectance mode. Melting points were measured using a Koffler bench. A ^57^Fe Mössbauer spectrum was recorded in transmission geometry mode with a constant acceleration mode conventional spectrometer equipped with a 50 mCi ^57^Co(Rh) source and a Reuter Stokes proportional counter. The powdered sample was sealed in aluminium foil, and the spectrum was recorded at room temperature. The spectrum was fitted using Recoil 1.05 Mössbauer Analysis software.^31^ The isomer shift values are given with respect to α-Fe at 298 K. Thermogravimetric Analyses (TGA) were carried out on a Mettler Toledo TGA/SDTA 851e analyser by loading 3–4 mg of sample, and the mass loss was monitored under nitrogen on warming from room temperature to 900 °C at 10 °C min^−1^. Magnetic susceptibilities were measured on a quantum design MPMS-5s SQUID magnetometer. The magnetic data were corrected for the sample holder and diamagnetic contributions. The crystal sample was quickly loaded into a gelatin capsule and immediately inserted within the SQUID cavity.

#### 2.1.1 Single crystal X-ray diffraction

Single-crystal X-ray diffraction data of 1–4 were collected on a MAR345 image plate detector using Mo Kα radiation at either ambient temperature or flash cooled to 150 K in a gaseous N_2(g)_ flow. The data were integrated with the CrysAlisPro software.^32^ The structures were solved by direct methods using the SHELXT program^33^ and refined by full matrix least squares on |*F*^2^| using SHELXL2014/7.^33^ The summary of data collection and crystallographic parameters of 1–4 are listed in Table S1.† Selected bond distances are given in Table S2.† CCDC 2073690–2073693 contain the supplementary crystallographic data for this paper.†

#### 2.1.2 Cell culture

Murin mastocytoma cell line P815 was cultured in RPMI 1640 medium supplemented with 5% heat-inactivated fetal bovine serum, 1% penicillin G-streptomycin, and 0.2% of l-glutamine. Incubation was performed at 37 °C in humidified atmosphere containing 5% CO_2_.

#### 2.1.3 Cell viability assay

Cellular viability was performed using the 3-(4,5-dimethylthiazol-2-yl)-2,5-diphenyltetrazolium bromide (MTT) assay.^34^ Briefly, the target cells (P815 murine mastocytoma) were washed twice with phosphate-buffered saline (PBS) and seeded on 96-well microtiter plates at the density of 10^4^ cells per well. Then, 100 μL of the culture medium supplemented with different concentrations (0–200 μM) of the compounds were added. Control cells were treated with DMSO alone. In all cases, the final concentration of DMSO never exceeded 0.2%. Following 48 h of incubation in humidified atmosphere at 37 °C and 5% CO_2_, 20 μL of MTT solution (5 mg mL^−1^ in PBS) was added to each well and incubated under the same conditions. After 4 h, 100 μL of medium was carefully removed from each well and replaced with 100 μL of acid–isopropanol (0.04 N HCl in isopropanol). The cell viability was measured in a spectrophotometer (MultisKan EX apparatus (Labsystem)) at *λ* = 540 nm and expressed as the percentage of control cells, using the following formula:% of cell viability = 100 × (*A*_i_/*A*_0_)where *A*_0_ and *A*_i_ are the optical density of control cells and treated cells, respectively. The cytotoxic activity of the compounds was compared by calculating the IC_50_ values (concentration leading to 50% of cell inhibition).

#### 2.1.4 Cytotoxic effect against human peripheral blood mononuclear cells (PBMCs)

This test was realized in order to evaluate the effect of the ligands and their active metal complexes against non-cancerous cells using the MTT colorimetric assay described below. To isolate the human PBMCs, blood samples in sterile heparinized tubes were collected under medical and ethical committee control from healthy volunteer donors. PBMCs were isolated using standard Ficoll-Hypaque density centrifugation. The interface lymphocytes were washed twice with phosphate buffer solution (PBS). The cytotoxic effect was measured in the same conditions and concentrations as detailed above for the tumor cells.

#### 2.1.5 Enzyme activity assays

##### (1) Preparation of cell extracts for antioxidant enzyme assays

P815 tumor cells were treated with the ligands and their active complexes for 48 h. Then, after washing once with PBS, the cells were harvested and centrifuged 12 000*g* for 10 min. The pellet was suspended in 500 μL of lysis buffer composed of 50 mM Tris–HCl, 1 mM phenylmethanesulfonyl (PMSF), 0.1% (v/v) Triton X-100, in 1.5 mL Eppendorf tubes and maintained in constant agitation at 4 °C for 30 minutes. The homogenate was then centrifuged (1600*g*, 20 min) at 4 °C. The supernatant (enzyme extract solution) was kept at −80 °C or used for the determination of superoxide dismutase (SOD), catalase, glutathione peroxidase (GPx), and glutathione reductase (GR) activities.

##### (2) Antioxidant enzyme assays

SOD activity was assayed according to the method of Sun *et al.*^35^ with some modifications. Briefly, the reaction mixture was composed of 0.05 M phosphate buffer, pH, 7.5, 10 mM methionine, 0.1 μM EDTA, 2 μM riboflavin, 75 μM Nitro Blue Tetrazolium (NBT) and the enzyme extract. The SOD activity was measured at 560 nm. One unit of SOD activity was defined as the quantity of SOD required to obtain a 50% inhibition of the reduction of NBT. The activity was expressed as units per mg of protein content.

The catalase activity was measured by the method of Aebi^36^ with some modifications. The final reaction volume of 0.5 mL included 0.1 M phosphate buffer, pH 7.0, 15 mM H_2_O_2_, and enzyme extract. The decomposition of H_2_O_2_ was monitored at 240 nm for 2 min at 25 °C. The extinction coefficient (43.6 M cm^−1^) was used to calculate the catalase activity, expressed as μmol of H_2_O_2_ decomposed per min per mg protein.

The GR activity was determined by the oxidation of NADPH at 340 nm as described by Carlberg and Mannervik.^37^ Briefly, the reaction mixture contained 0.1 M phosphate buffer, pH 7.6, 1 mM GSSG and 0.2 mM NADPH. The contents were incubated at 25 °C for 3 min and the reaction was initiated by adding enzyme extract. GR activity was expressed as nmol of NADPH oxidized per min per mg of protein by using the extinction coefficient of 6.2 mM^−1^ cm^−1^.

GPx activity was carried out according to the method described by Lawrence & Burk^38^ with some modifications. The reaction mixture contained 0.1 M potassium phosphate, pH 7.0, 1 mM EDTA, 1 mM sodium azide, 1 mM GSH, GR (10 μg mL^−1^), 0.25 mM NADPH and enzyme extract. The mixture was incubated at 25 °C for 3 min and completed by adding 0.25 Mm of H_2_O_2_. The rate of NADPH oxidation was monitored at 340 nm for 5 min. GPx activity was calculated and expressed as μmol of NADPH oxidized per min per mg of protein by using the extinction coefficient of 6.2 mM^−1^ cm^−1^.

The total soluble protein content of the enzyme extracts was determined following the method of Bradford,^39^ using Bovine Serum Albumin (BSA) as a protein standard.

### 2.2. Syntheses

**Scheme 2:**
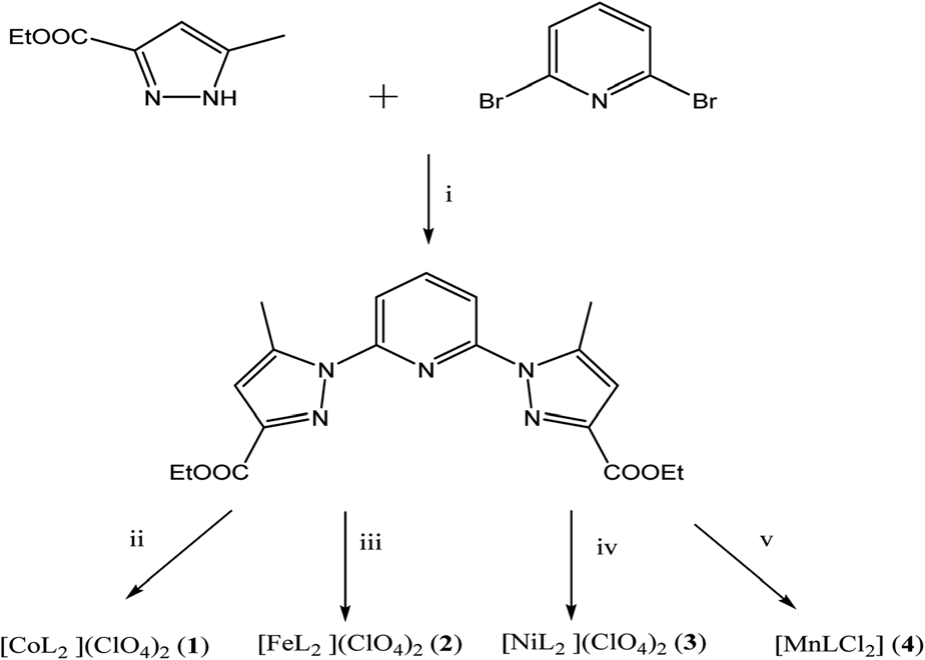
Synthetic routes towards L and 1–4. (i) tBuOK, Ar_(g)_, DMSO extra dry (ii) Co(ClO_4_)_2_·6H_2_O. (iii) Fe(ClO_4_)_2_·6H_2_O. (iv) Ni(ClO_4_)_2_·6H_2_O. (v) MnCl_2_·6H_2_O.

The synthesis of L first involves the synthesis of pyrazolic ester 2-ethyl 5-methyl-1*H*-pyrazole-3-carboxylate (L_pyz_), which is carried out in two steps, as reported previously by our group (Scheme 2).^15^ The synthesis protocol and characterization of L′, 1′ and 2′ have been described in our previous work.^30^

#### 2.2.1 Synthesis of diethyl 1,1′-(pyridine-2,6-diyl) bis(5-methyl-1*H*-pyrazole-3-carboxylate) (L)

The synthesis of L was carried out adapting a literature method.^40^ An excess of L_pyz_ (13.66 g, 0.09 mol) and potassium *tert*-butoxide (9.94 g, 0.09 mol) were both dissolved under Ar_(g)_ at r.t. in dry and degassed DMSO 99.8% (20 mL) to give a dark red solution. The mixture was stirred for 30 min, then dibromopyridine (7 g, 0.03 mol) solubilized in DMSO 99.8% (10 mL) was added. The mixture was kept under Ar_(g)_ for 24 h at 140 °C. After the reaction time has elapsed, it was cooled to r.t. and filtered. A white precipitated was formed by adding water (50 mL), which was filtered and washed with water (3 × 20 mL). Yield 42% (4.82 g). Mp = 125(1) °C. FT-IR/cm^−1^: 2985 (w), 1724 (s), 1601 (m), 1427 (s), 1243 (s), 810 (m). ^1^H NMR [300 MHz, CDCl_3_] *δ* (ppm) = 2.62 (t, 6H, CH_3_–CH_2_); 2.62 (s, 6H, CH_3_pyrazolyl); 4.43 (q, 4H, CH_2_–O); 6.74 (s, 2H, CH_pyrazolyl_); 7.93 (m, 3H, pyridine). ^13^C NMR [300 MHz, CDCl_3_] *δ* (ppm) = 13.97 (2CH_3,pyrazolyl_), 14.79 (CH_3_–CH_2_), 61.18 (CH_2_–O), 111.06 (CH_pyrazolyl_), 117.35–142.63 (pyridine), N=C_pyz_(150.89). MS (ESI), *m*/*z*: 406.1483 [MNa^+^].

#### 2.2.2 Synthesis of [CoL_2_](ClO_4_)_2_ (1)

A solution of Co(ClO_4_)_2_·6H_2_O (18.2 mg, 0.05 mmol) and L (38.3 mg, 0.1 mmol) in MeOH (10 mL) was stirred for 15 min. The solution was left at room temperature. The obtained powder was recrystallized by slow diffusion of diethyl ether (30 mL) into the methanolic solution. Pink needle crystals of 1 suitable for X-ray measurements were filtered off after 10 days. Yield 23% (12 mg). FT-IR/cm^−1^: 3134 (m), 2989 (w), 1623 (m), 1235 (s), 856 (w). HRMS (ESI): *m*/*z*: 924.1997 [L_2_^35^ClO_4_^59^Co].

#### 2.2.3 Synthesis of [FeL_2_](ClO_4_)_2_ (2)

A solution of Fe(ClO_4_)_2_·6H_2_O (18.1 mg, 0.05 mmol) in acetone (10 mL) was added to a solution of 10 mL of L (38.3 mg, 0.1 mmol) in chloroform (20 mL), and the mixture was stirred for 15 min. An orange/yellow solution was formed. A yellow powder was obtained by slow addition of diethyl ether (40 mL) into this solution. After 30 min stirring, the solution was filtered and the residue was dissolved in nitromethane (6 mL), and vapour diffused with Et_2_O. Diffusion was complete in about 2 weeks and X-ray quality yellow crystals were collected by filtration. Yield 31% (16 mg). FT-IR/cm^−1^ 3134 (w), 2994 (w), 1635 (m), 1235 (s), 856 (w). HRMS (ESI): *m*/*z*: 921.2016 [L_2_^35^ClO_4_^56^Fe].

#### 2.2.4 Synthesis of [NiL_2_](ClO_4_)_2_ (3)

A solution of Ni(ClO_4_)_2_·6H_2_O (36.5 mg, 0.10 mmol) in methanol (7 mL) was added to a solution of L (19.1 mg, 0.05 mmol) in nitromethane (10 mL) and the mixture was stirred for 10 min. To this blue solution, diethyl ether (30 mL) was slowly diffused to obtain blue needle crystals suitable for X-ray diffraction. Yield: 24% (12.1 mg). FT-IR/cm^−1^ 2985 (w), 1731 (m), 1596 (m), 1240 (s), 856 (w). HRMS (ESI): *m*/*z*: 923.2020 [L_2_^35^ClO_4_^58^Ni].

#### 2.2.5 Synthesis of [MnLCl_2_] (4)

15 mL of acetonitrile solution of MnCl_2_·4H_2_O (9.8 mg, 0.05 mmol) and L (38.3 mg, 0.1 mmol) in a ratio 1 : 2 was stirred at r.t. for 16 h then concentrated. The obtained powder was solubilised in nitromethane (6 mL) and recrystallized by slow diffusion of diethyl ether (30 mL) at r.t. After 10 days, white single crystals were obtained. Yield: 38% (9.5 mg) FT-IR/cm^−1^ 3150 (w), 2964 (w), 1737 (s), 1627 (m), 1442 (s), 856 (w). HRMS (ESI): *m*/*z*: 473.0655 [L^35^Cl^55^Mn].

## 3. Results and discussion

### 3.1. Synthesis and characterisation

L has been prepared by nucleophilic aromatic substitution reaction of pyrazolic anions with 2,6-dibromopyridine (Scheme 2). Given the low reactivity of aryl halides and the poor nucleophilicity of even unhindered pyrazole anions, these reactions require rather severe conditions. According to literature, these reactions are quite sensitive to steric hindrance, with more hindered 3,5-disubstituted pyrazole anions reacting very poorly with pyridyl halides even under stringent conditions.^41^

**Scheme 3:**
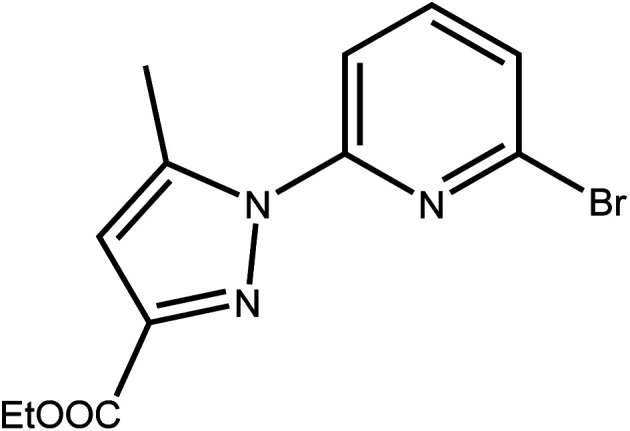
The monosubstituted intermediate ethyl 5-methyl-1-(pyridin-2-yl)-1*H*-pyrazole-3-carboxylate 2′.

The most common impurity, when unsymmetrically substituted pyrazole is used, is the monosubstituted intermediate 2′,^41^ shown in Scheme 3 which is hard to isolate. Despite various tests, by increasing temperature, reaction time or even changing the solvent or the base, this type of ligand has shown resistance in its synthesis.

**Scheme 4:**
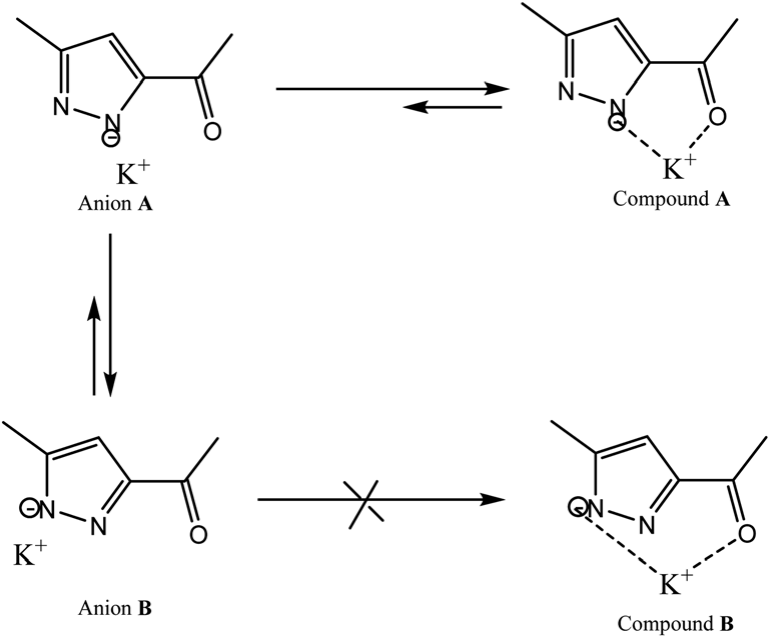
Stabilization of pyrazolate anion through K^+^ cation.

Therefore, we have abandoned these classic processes and we carried out this reaction under an inert atmosphere, using Ar_(g)_ and dimethyl sulfoxide extra dry as solvent at very high temperature. We point out that the use of 3(5) pyrazole substituted asymmetrical pyrazole possessing an ester function could, by its particular electronic effects, induces the orientation of the alkylation on the α-nitrogen.^42^ Furthermore, the use of potassium *tert*-butoxide as a base, acts as a strong base and weak nucleophile, thus leading to the stabilization of β-nitrogen pyrazolate anion through the K^+^ cation (compound A), and to the decrease of its free energy relative to that of α-nitrogen pyrazolate anion (compound B) (Scheme 4). This implies that the activation energy required to reach the transition state is lower in the case of compound B compared to compound A. Therefore, compound B will more readily leads to the alkylation product than compound A. This explains why attack on the α-nitrogen leads to the majority product. Therefore, the pyrazolate anion reacts selectively at the nitrogen atom adjacent to the methyl group. The target product L was confirmed by FT-IR, ^1^H NMR, ^13^C NMR and high-resolution mass spectrometry (HRMS).

### 3.2. Structural characterization

**Fig. 1:**
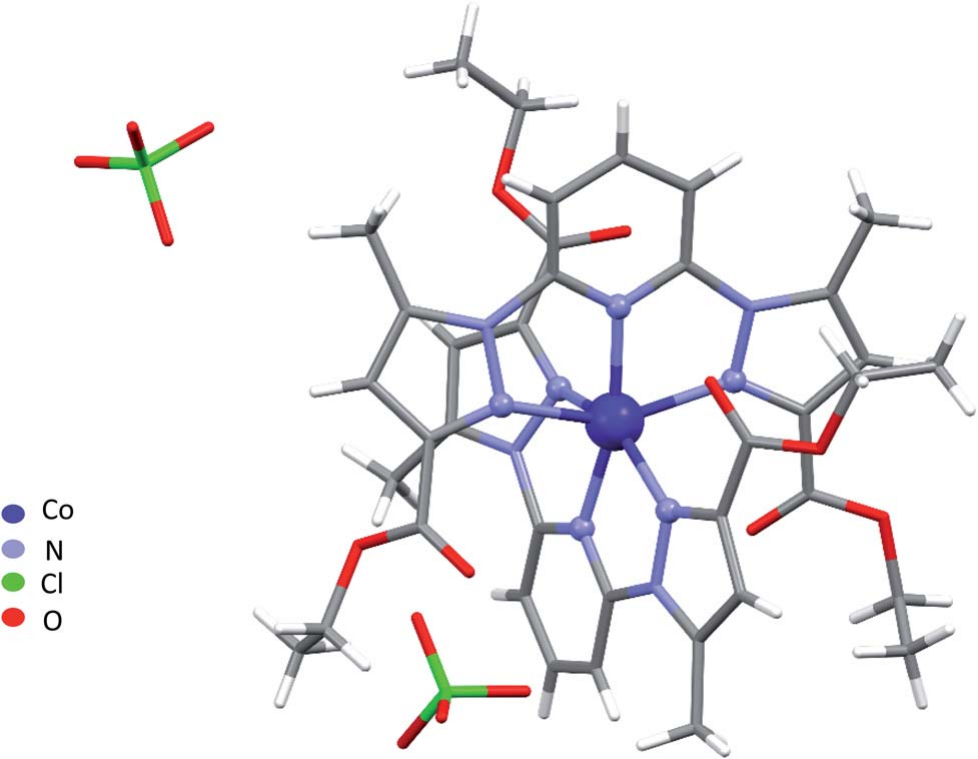
Perspective view of the molecular structure of 1. Disordered parts were omitted for clarity.

**Fig. 2:**
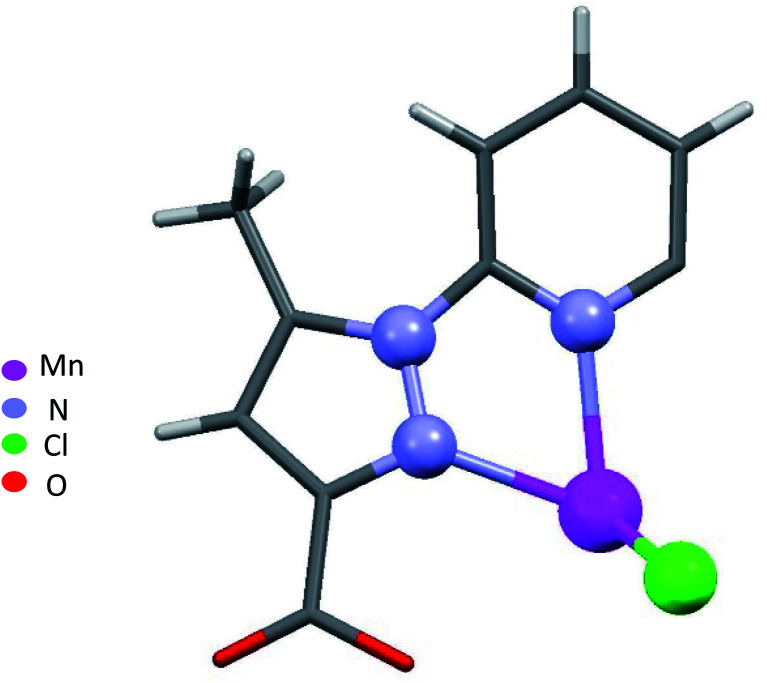
Asymmetric unit of 4.

Single-crystal X-ray analysis revealed that 1–4 are mononuclear coordination complexes (Fig. 1 and 2), L acting as a tridentate chelator, with the pincer uniquely in a meridional *mer* coordination with an overall planar skeleton (Fig. S1 and S2†). In Co(ii), Fe(ii) and Ni(ii) complexes the presence of weakly coordinating ClO_4_^−^ anions, do not play a big role in stabilizing the crystal packing as they were mostly found to be disordered. Nevertheless, the double interaction between a carbonyl oxygen and the twisted pyrazolyl ring centroids (Fig. S3†) leads in 1 and 2 to a propagation of this interaction throughout the crystal packing. While in 3, this propagation is disrupted as only one ligand is significantly twisted. Similar contacts between a carbonyl oxygen and a pyrazole ring are not so common, CSD searches only show about 400 structures with this motive. The closely related structure (CCDC refcode GAPTEX^43^) also displays this double interaction.

The overall molecular structure of complexes 1–3 is quite similar with a maximal root-mean-square deviation (RMSD) of 0.89 Å, taking all atoms in account (Fig. S4–S6†). The *mer* coordination mode of the tridentate ligands necessarily places the pyridyl nitrogen atoms in axial position, with four nitrogen of four pyrazolyl groups from two ligands L in the equatorial plane, to form an octahedral geometry around the central metal atom. The ligands are therefore located on opposite sides of the metal centre with a dihedral angle between the ligand planes (calculated through the aromatic rings of the ligands) of 67.09°, 59.97°, and 75.75° for 1, 2, and 3 respectively. Table S2† lists the bond lengths and bond angles around the metal atom for complexes 1–3.

A large torsion angle is observed between one pyrazolyl ring and the central pyridyl ring in complexes 1, 2 and 3. Both angles being 23.4(3)°, 19.8(3)° and 21.9(4)°, 20.6(4) ° in 1 and 2, respectively and 24.5(2)° in 3 which is in line with the values from complexes 1 and 2, and 9.8(2), which is significantly less. Furthermore, the torsion angle bends both pyrazolyl rings of both ligands towards each other, bringing both rings over each other, with a longer inter-centroid distance 4.112 Å in the Ni(ii) complex followed by 3.857 Å in the Co(ii) and 3.500 Å in the Fe(ii) complex (Fig. S7†). The EtO groups on the same side were found to be disordered and were refined over two discrete positions, except for the Ni(ii) complex for which no disorder was observed during structure refinement.

NNN pincer ligands forming an octahedral structure are characterised by their bite angle, resulting in a significant octahedral distortion. In this study, the bite angle for 1–3 lies in the range 143–153°, leading to a strong variation of the distortion parameter *Σ* from 124.93° in complex 3, through 149.37° in complex 1, to 172.86° in 2. We recall that *Σ* measures local angular distortions of the octahedral donor set, where *α*_*i*_ are the 12 *cis*-N–Fe–N angles at the metal centre^44^ following eqn (1):1
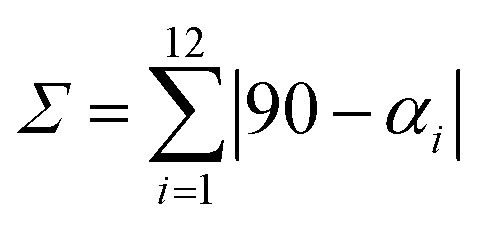


These values are well compared to reported Co(ii) and Ni(ii) complexes with bpp ligands.^45–47^ Iron complex 2 expected to be high-spin (HS) at room temperature, as earlier noticed for Fe(ii) complexes with NNN tridentate ligands with narrow bites like bpp or terpyridine.^48,49^ Indeed, the Fe–N bond length of 2.18 Å (Table S2†) indicates a HS state. The coordination geometry of 2 is significantly distorted from the ideal *D*_2d_ symmetry. This angular distortion involves a decrease in N(pyridine)–Fe–N(pyridine) angle from its optimal value 180° to 157.6(1)° and twisting of the two planes defined by the tridentate ligands away from the perpendicular 90° decreasing to 59.97°, which is characteristic of an angular Jahn–Teller distortion. Two parameters are discussed: *Σ* and *Θ* which defines more specifically the degree of trigonal distortion of the coordination geometry from an octahedron towards a trigonal prism (eqn (2)):2
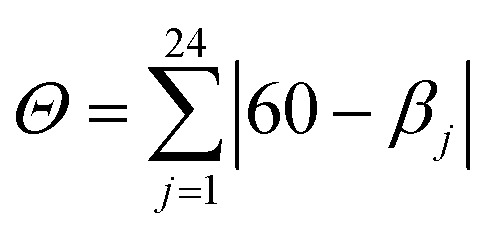
where *β*_*j*_ are the 24 unique torsion angles between adjacent N donors on opposite triangular faces of the octahedron, measured along their common (pseudo)-three-fold axis.^50^ An ideal octahedron would afford zero distortion parameters. Herein, we compare *Θ* and *Σ* values in other HS and LS iron(ii) bis (pyrazolyl)pyridine (bpp) complexes (Table 1).

**Table 1:** Distortion parameters for HS and LS Fe(ii) bpp complexes^a^

Complex	Spin state	*Θ*	*Σ*	Reference
[FeL_2_](ClO_4_)_2_	HS – distorted structure	670	172.86 (1)	This work
[Fe(1-bpp)_2_](PF_6_)_2_	HS at all T – distorted structure	559	197.2 (2)	51
[Fe(1-bpp)_2_](ClO_4_)_2_	HS at all T – distorted structure	547	186.6 (2)	43
[Fe(L^1^)_2_](SbF_6_)_2_	HS at all T – undistorted structure	460	148.8 (4)	52
[Fe(1-bpp)_2_](BF_4_)_2_	Spin crossover on cooling	467	150.8 (2)	53
[Fe(L^3^)_2_](ClO_4_)_2_	LS at 30 K	288	89 (1)	48

a 1-bpp = 2,6-di(1*H*-pyrazol-1-yl)pyridine; L^1^ = 2,6-bis(3-methyl-1*H*-pyrazol-1-yl)pyridine; L^3^ = (2,6-di(1*H*-pyrazol-1-yl)pyridin-4-yl)methanol.

It is clear that there is a strong correlation between distortion parameters *Θ* and *Σ* and the spin state of the metal complexes. Indeed, octahedra of LS iron(ii) ions show less variation than HS complexes, as a consequence of their more regular geometry. Iron centre 2 remains HS on cooling, which is consistent with our magnetic study (*vide infra*).

This behaviour is due to the rigid lattice and to steric hindrance of 2 which prevents any SCO to occur, since a rigid lattice cannot accommodate the resulting structural changes,^54^ revealed by the contraction of the Fe–N bonds required for the LS state as observed in earlier examples.^55^

When chloride is used instead of perchlorate, another geometry is obtained [MnLCl_2_] (4) (Fig. 2). The Mn(ii) ion is penta-coordinated by one trigonal pincer ligand L using two pyrazole nitrogen and one pyridine N atom, the two remaining sites are occupied by two monodentate chloride counter anions.

**Fig. 3:**
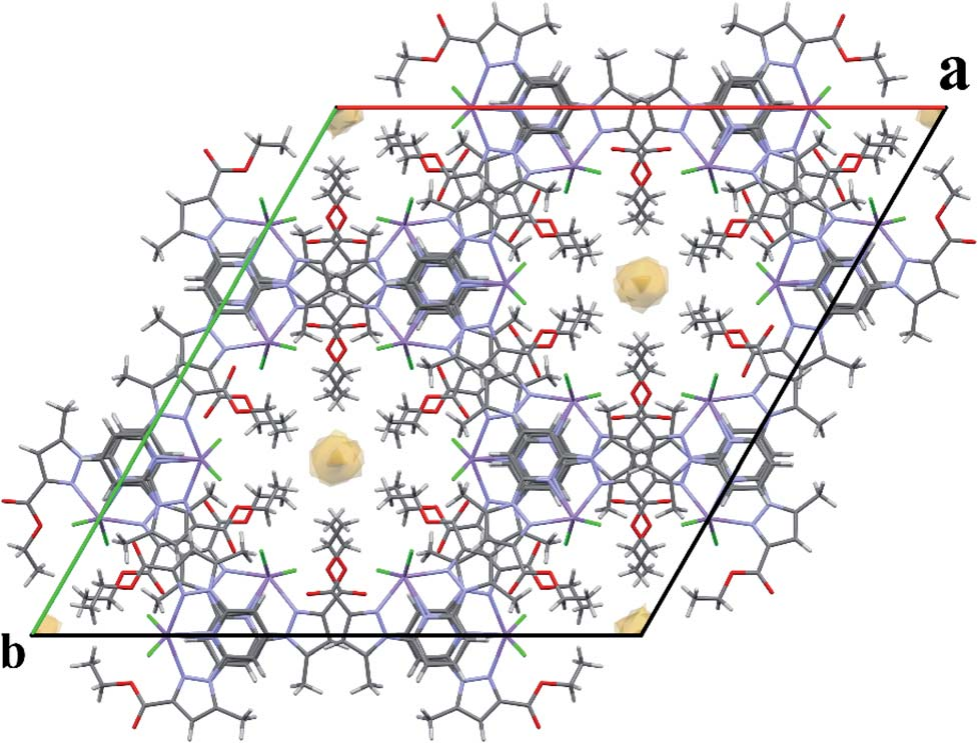
Expanded unit cell of 4, showing the hydrophobic channel along the *c*-axis. Disordered parts of the ethyl chains are omitted for clarity.

For a pentacoordinated metal centre, the distortion of the coordination environment can be rated by the Addison distortion index *τ*_5_ = (*α* − *β*)/60, where *α* and *β* are the two largest coordination angles^56^ (*τ* = 0.00 for square pyramid (SP) and 1.00 for trigonal bipyramidal (TP)). In our case, the Addison distortion index *τ*_5_ = 0.07; indicates that the Mn complex has a nearly perfect square pyramid geometry, with one Cl atom in the axial position. Table S3† lists the bond lengths and angle values of complex 4. These observed geometrical features are quite comparable to reported Cd(ii) complexes with *τ*_5_ = 0.046.^57^ Furthermore, complex 4 is located on a 2-fold axis, running though the pyridyl ring and the Mn atom, with the asymmetric unit being half a formula unit in space group *R*3̄. During structure refinement the pyridyl ring was found slightly off the 2-fold axis and disorder was modelled for the central ring of the ligand. Also, here the ethyl groups are disordered and refined over two sites. In the crystal packing all disordered ethyl groups are pointing towards the crystallographic 3-fold axis, forming a hydrophobic channel of alkyl chains (Fig. 3).

### 3.3. Fourier transform infrared spectroscopy (FT-IR)

The vibrational structure of all complexes with their ligand were characterized by FT-IR using KBr discs on a PerkinElmer 1310 spectrometer and recorded from 4000 to 400 cm^−1^. In all spectra 1–4 and L (see ESI†), three characteristic adsorption bands are observed: the first one around 3000 cm^−1^ characteristic of aromatic C–H elongation vibration in both pyridine and pyrazolyl rings. The wide and strong bands between 1730–1750 cm^−1^ are attributed to the absorption of the asymmetric and symmetric stretching vibration of the C=O from the ester function. The third characteristic adsorption bands appearing at 1595 and 1612 cm^−1^ were assigned to C=N imine vibrations of pyrazole and pyridine groups. The comparison of the IR spectra of the ligand to its complexes indicate that the imine band is shifted up by 17 cm^−1^ in complex 2 and shifted down by 7 cm^−1^ in complex 1. It testifies the reactivity of this function where the metal was coordinated to the ligand through imine groups as tridentate pincer ligand. Furthermore, we notice the appearance of a new band at low frequency 632 cm^−1^ only in the spectra of the iron metal complex and cobalt complexes, which refers to non-coordinated perchlorate anions.^58^ Whereas, in manganese complex the signal around 200 cm^−1^ specific to chlorine–metal–ligand bending,^59^ is absent since it is not covered by the 400–4000 cm^−1^ range studied.

### 3.4. Diffuse reflectance spectroscopy (DRS)

**Fig. 4:**
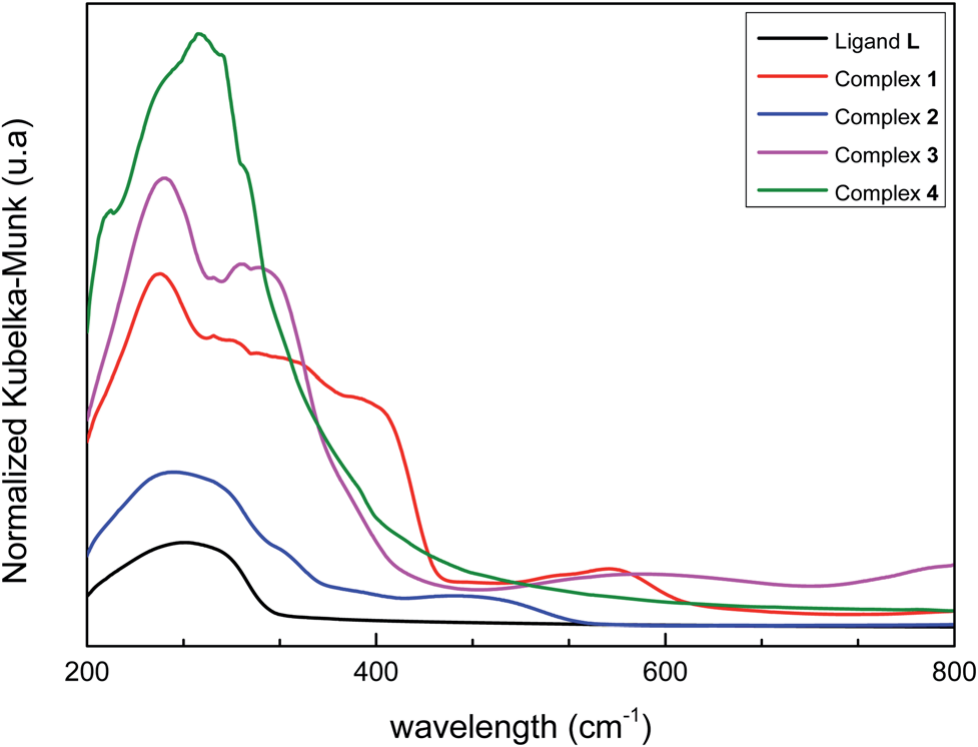
Normalized diffuse reflectance spectra of L and 1–4.

Solid-state UV/vis spectra of L and its coordination complexes 1–4 are displayed in Fig. 4. An examination of this figure indicates that the metal ion is well coordinated to the ligand. The free ligand L itself shows only one weak absorbance at *λ* = 270 nm arising from the π–π* transition of the aromatic rings. As can be noticed, this band is not strongly perturbed in the Co(ii), Fe(ii), Ni(ii) and Mn(ii) complexes suggesting that coordination of the metal ions hardly alters the intrinsic electronic properties of the ligand.^60^ Additional bands are observed in the UV region for compounds 1–4, which can be assigned to metal-to-ligand charge transfer (MLCT) processes. However, weak bands in the visible region at *λ* = 563 nm, 473 nm and 589 nm are observed respectively in cobalt, iron and nickel complexes corresponding to d–d transition. In 1, a broad band is observed, which presumably originates from the association of molecules though intermolecular hydrogen bonding.^61^

### 3.5. Powder X-ray diffraction (XRD) and thermogravimetric analysis

Powder X-ray diffraction (XRD) patterns of 1–4 at room temperature well match the diffractograms simulated from the single-crystal X-ray diffraction data, thus confirming a phase purity of the bulk samples (see ESI†). The thermal behaviour and stability of compounds L and 4 was studied by thermogravimetric analysis from room temperature to 900 °C under a N_2(g)_ atmosphere. Complexes 1, 2 and 3 were not studied since their structures include perchlorate ions, which are potentially explosive.^62^ The thermogravimetric analysis plot of the ligand L (see ESI†) shows one mass loss of 96.5% observed between 200 and 380 °C, corresponding to the loss of two pyrazole rings and of the pyridine ring (theo. for C_19_H_24_N_4_O_4_: 96.2%), thus demonstrating its relative high thermal stability. The thermal decomposition of complex 4 takes place in three steps (see ESI†). The first decomposition step appears between 195–390 °C with a mass loss of 49.3% (theo. 50.3%) corresponding to the degradation of part of the pyrazole skeleton loosing two C_7_H_12_O_2_ units. The second step occurs within the temperature range 380–660 °C and represents the volatilization of two Cl anions and four pyrazole's nitrogen with mass loss of 23.7% (theo. of 24%). The last step above 660 °C was assigned to pyridine pyrolysis.

### 3.6. ^57^Fe Mössbauer spectroscopy

**Fig. 5:**
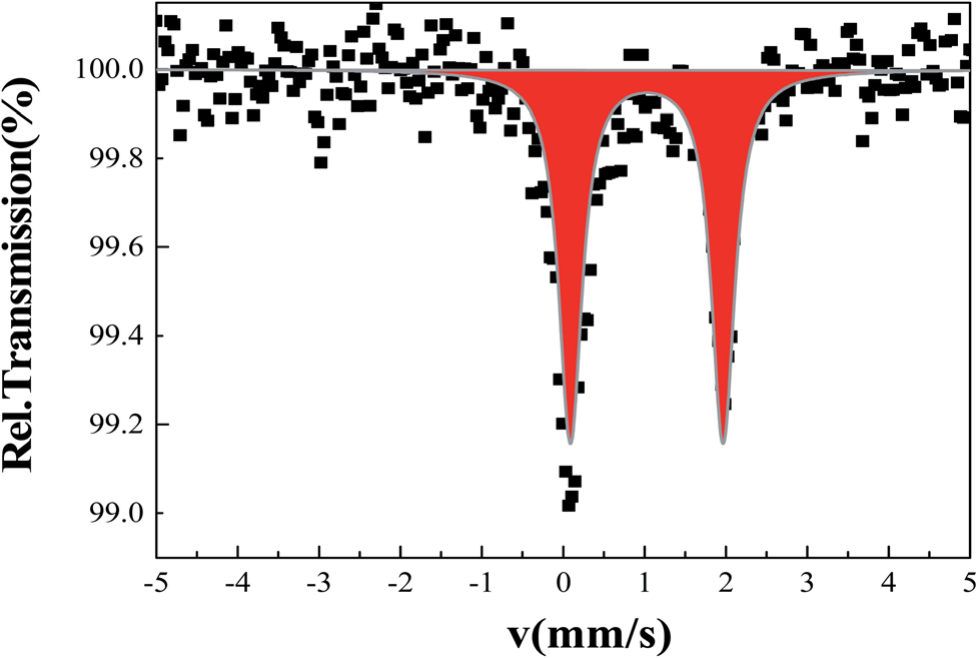
Room temperature Mössbauer spectrum for single crystals of 2.

A Mössbauer spectrum of 2 as single crystals recorded at room temperature is shown in Fig. 5. The data were fitted by least square refinement. As a result, a unique quadrupole doublet is observed, with isomer shift *δ* = 1.02(1) mm s^−1^ and quadrupole splitting Δ*E*_Q_ = 1.87(3) mm s^−1^. These parameters are characteristic of HS iron(ii) bpp complexes. The half-width at half-maximum was *Γ*/2 = 0.17(2) mm s^−1^. The observed asymmetry of the lines is due to a texture effect, while measuring single crystals. Such asymmetry was not taken into account in the current fit. While doing so, a fraction of Fe(iii) species were detected as a result of the long acquisition in air of the spectrum.^63^ No colour change was observed on cooling to liquid nitrogen suggesting that 2 remains in the HS ground state. This will be confirmed in the next section by recording temperature dependent magnetic susceptibility data.

### 3.7. Magnetic properties

The temperature dependence of *χ*_M_*T*, *χ*_M_ being the molar magnetic susceptibility for 2, was investigated over the range 4–400 K (Fig. S8†). At room temperature and above, *χ*_M_*T* = 3.7(1) cm^3^ K mol^−1^, in agreement with the HS state of iron, as detected by Mössbauer spectroscopy (Fig. 5). No spin state crossover is detected be on cooling, or on warming above room temperature. Such a HS state fits with the large distortion parameters and Fe–N distances (Table S2†). The drop in *χ*_M_*T* below 25 K is due to zero-field splitting of HS Fe^II^ ions.

### 3.8. Biological activity

#### 3.8.1 Cell viability

We have investigated the biological activity of L and its coordination complexes 1–4 as well as the one of L′, [MnL′Cl_2_] (1′), [CdL′Cl_2_] (2′). The *in vitro* antitumor activity was determined by the measurement of their cytostatic and cytotoxic properties towards the tumor cell line P815 using the MTT assay.

**Fig. 6:**
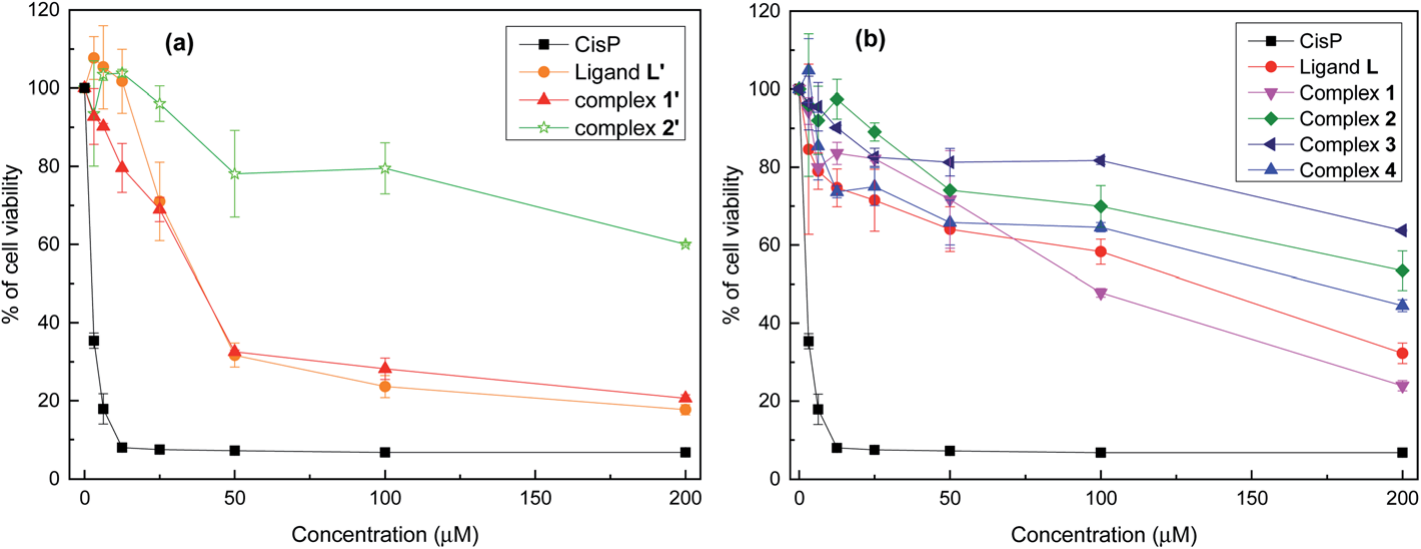
Viability of P815 mastocytoma cells after treatment for 48 h with different concentrations of L′ (a) and L (b) derivatives, evaluated by MTT assay. Data are means ± SD of representative of three independent experiments.

Fig. 6 shows the effect of increasing concentrations of each compound on the viability of P815 tumor cells. As can be seen, all the compounds showed a concentration dependent effect but remarkably L′ and 1′ showed the highest antitumor activity, as well as a fast decrease up to 50 μM.

Table 2 shows the IC_50_ values which were determined for each compounds, taking *cis*-[Pt(NH_3_)_2_Cl_2_] (CisPt) as a positive control with IC_50_ = 1.42 ± 0.035 μM. L′ and 1′ exhibit a potent cytotoxic activity with IC_50_ = 35.12 μΜ and 34.77 μM, respectively. However, L, 1 and 4 display a moderate cytotoxic activity, whereas 2′, 2 and 3 show a lower cytotoxic activity with IC_50_ values higher than 200 μM. Given together, the eight compounds can be classified on the basis of their cytotoxic level against the P815 cell line in the following order: 1′ ≥ L′ > 1 > L > 4 > 2 > 2′ > 3.

**Table 2:** Inhibitory concentration 50 (IC_50_) in μM of L′ and L derivatives tested against P815 tumor cell line^a^

	Molecules series								
	L′	1′	2′	CisPt	L	1	2	3	4
IC_50_ (μM)	35.12 ± 3.71^a^	34.77 ± 1.09^a^	>200	1.42 ± 0.035^b^	132.8 ± 8.2^c^	112.41 ± 3.41^c^	>200	>200	173.52 ± 7.46^d^

a Values are means standard deviation for at least six determination. Different letters indicate significant difference (*p* < 0.05) within conditions according to Tukey's multiple comparison test.

Thus, the cytotoxicity of L′ is much higher than the one of L. Such activity is most likely due to the conformation of C–C pyridine–pyrazole bond which is rigid, stable and likely bioactive. In contrast to the C–N pyridine–pyrazole bond, found in L, which exhibits greater flexibility suggesting that the ligand passes through different conformations. It is known that conformational stability is a determinant of drug cytotoxicity.^64^ Bioactive conformation is one of the important pharmacophore which is defined by the IUPAC as “an ensemble of steric and electronic features that is necessary to ensure the optimal supramolecular interactions with a specific biological target and to trigger (or block) its biological response”.^65,66^ On the other hand, the improvement in activity is also due to the complexation metal. Interestingly the cytotoxicity activity of L′ was maintained in 1′ but decrease considerably when substituting Mn(ii) by Cd(ii) for 2′. The manganese complexes (1′ and 4) have considerably better activity than the other complexes, in their respective series, especially the Mn complex formed with the C–C type ligand (L′). This is not surprising given that tumor-targeting manganese complexes induce reactive oxygen species (ROS)-mediated apoptotic and autophagic cancer cell death.^67^

#### 3.8.2 Cytotoxicity against PBMCs as normal cells

**Fig. 7:**
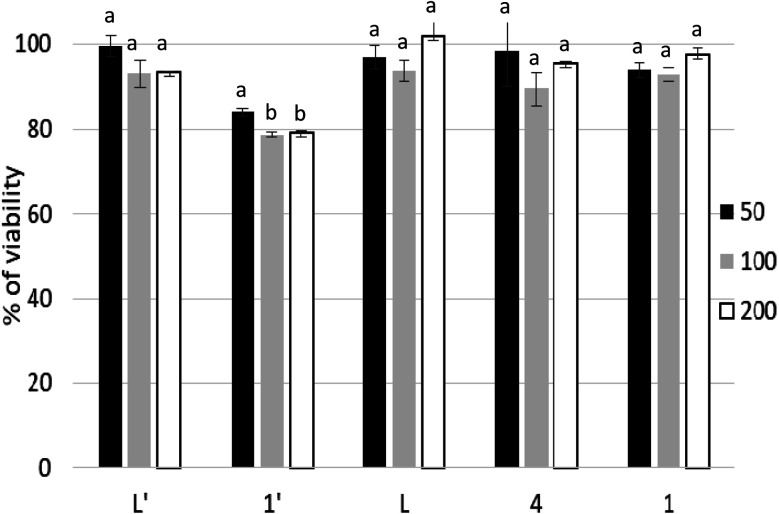
Viability of PBMCs treated with ligands L, L′ and their metal complexes (1–4, 1′, 2′) for 48 h. Cell viability was calculated as described in materials and methods. Data represented as mean of three independent measurements. Bars represent the standard deviation. Different letters in the same column indicate significant differences (*p* < 0.05) within conditions according to one-way ANOVA multiple comparison range test.

Peripheral Blood Mononuclear Cells (PBMCs) are the first normal cell populations that come into contact with antitumor drugs used in conventional chemotherapy and that collapses from the first week of intravenous treatment of patients resulting in significant immune deficiency and increased side effects.^68^ In this study, we have tested our compounds towards tumor cells, against human PBMCs from healthy donors in order to determine their effects against normal cells. Interestingly, ligands and their active metal complexes did not show a significant cytotoxicity towards human normal PBMCs (Fig. 7). These findings suggest a selective killing ability of these molecules against P815 cells without impacting normal cells.

#### 3.8.3 Antioxidant enzyme activity

The main objective of this study is to better understand the mechanisms of our compounds in the protection of P815 cell line against stress. Many studies have reported that cancer cells have an elevated level of ROS compared to normal cells.^69^ However, the elevation of ROS to a certain level may be lethal for tumor cells themselves. As a normal strategy from cells to face the oxidative stress, they increase the levels of antioxidant enzyme's activity, where SOD is recognized as the primary defense barrier against ROS by catalyzing the dismutation of superoxide anion radicals (O_2_˙^−^) to hydrogen peroxide (H_2_O_2_). H_2_O_2_ generated by the activity of SOD is eliminated by its conversion to H_2_O in subsequent reactions by CAT and GPx.^69^ Accumulation of H_2_O_2_ in the cell was reported to be implicated in the induction of apoptosis.^70^ The intracellular antioxidant capacity is mainly also conferred by glutathione (GSH) dependent systems. In fact, the important enzymes implicated in the regulation of redox homeostasis such as glutathione reductase are limited to the pool of GSH as a source of reducing equivalents.^71^

**Fig. 8:**
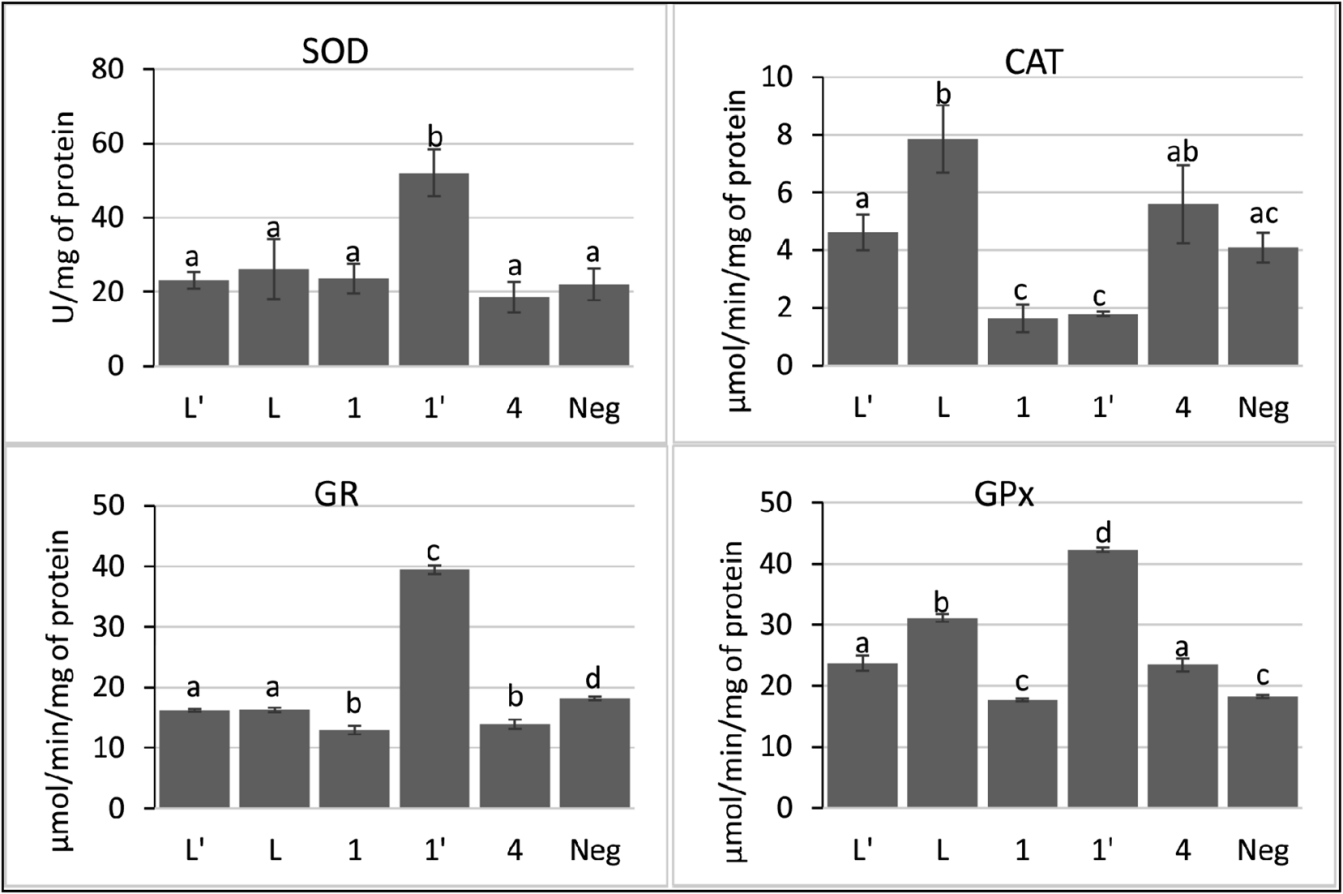
Activities of antioxidant enzymes; superoxide dismutase (SOD), catalase (CAT), glutathione peroxidase (GPx), and glutathione reductase (GR) in P815 cell line after treatment with ligands L, L′ and their metal complexes (1–4, 1′, 2′). Each value represents the mean of at least four replicates. Bars represent the standard deviation. Different letters in the same column indicate significant differences (*p* < 0.05) within conditions according to one-way ANOVA multiple comparison range test.

In the current study, we show that P815 tumor cells exposure to studied compounds differentially affects the SOD, CAT, GPx and GR activities. According to Fig. 8, the metal complex 1′ had the ability to increase selectively and significatively the activity of SOD, GPx and GR, compared to negative control and other tested compounds, as a defending strategy of cells face to the oxidative stress to decrease the ROS. However, compounds L, L′, 1 and 4 did not affect significatively the activity of SOD, while inducing significant decrease in GR activity. In addition, these compounds affect differentially CAT and GPx activities; L, L′ and 4 induced a small increase in the activities of GPx. However, L has the ability to increase the activity of CAT.

Taken together, these findings suggest that 1′ acts through the induction of intracellular oxidative stress. In addition, 1′ may induce the accumulation of H_2_O_2_ by its ability to inhibit the catalase activity. The other compounds seem to modulate the pool of GSH by decreasing the activity of GR.

## 4. Conclusions

This study has investigated synthesis, structural chemistry, cytotoxic and antioxidant activity of a series of [M(bispyrazolyl pyridine)]^2+^ derivatives belonging to C–N junction, bearing sterically substituents with Co(ii), Fe(ii), Ni(ii) and Mn(ii). Our results have shown mononuclear structures promoted by tridentate ligand *via* two pyrazolyl N-atoms and one N-pyridine donor atom to set up distorted octahedra for Co^2+^, Fe^2+^, Ni^2+^ and a distorted square-pyramidal geometry for Mn^2+^. Moreover, L′ (C–C junction) and its Mn(ii) 1′ complex exhibited better antitumor activity compared to the other compounds, also all cytotoxic products are safe on normal cells PBMCs. 1′ stimulate SOD and inhibit CAT, this inhibition induces the accumulation of H_2_O_2_ in the cell, that could implicate the induction of apoptosis and consequently cell death. As a conclusion, this study highlighted the strong correlation between pyridine–pyrazolyl rings junction and anticancer activity, however further investigation is required in this important research area.

## Conflicts of interest

There are no conflicts to declare.

## Supplementary Material

RA-011-D1RA05918A-s001

RA-011-D1RA05918A-s002
