# Supplementary material for: Synthesis and cytotoxicity against tumor cells of pincer N-heterocyclic ligands and their transition metal complexes
Source: RSC Adv. 2021 Oct 27;11(55):34742–53. doi: 10.1039/d1ra05918a (PMC9042687; doi:10.1039/d1ra05918a)
Supplement: RA-011-D1RA05918A-s001 [file RA-011-D1RA05918A-s001.pdf]

Electronic Supplementary Material (ESI) for RSC Advances.
This journal is © The Royal Society of Chemistry 2021

Electronic Supplementary Information (ESI)

Synthesis and Cytotoxicity against Tumor Cells of Pincer *N*-heterocyclic Ligands and their Transition Metal complexes

Afaf Oulmidi[1,2], Smaail Radi[1,*], Abderrazak Idir[3], Zyad Abdelmajid[3], Imad Kabach[4], Mohamed Nhiri[4], Koen Robeyns[2], Aurelian Rotaru,[5] and Yann Garcia[2,*]

[1] LCAE, Department of Chemistry, Faculty of Sciences, University Mohamed I, BP 524, 60 000 Oujda, Morocco;

[2] Institute of Condensed Matter and Nanosciences, Molecular Chemistry, Materials and Catalysis (IMCN/MOST), Université catholique de Louvain, Belgium;

[3] Team of Experimental Oncology and Natural Substances, Cellular and Molecular Immunopharmacology, Faculty of Sciences and Techniques, Sultan Moulay Slimane University, Mailbox 523, 23000 Beni Mellal, Morocco;

[4] Laboratory of Biochemistry and Molecular Genetics, Faculty if sciences and Technology, Tangier, Morocco;

[5] Department of Electrical Engineering and Computer Science and MANSiD Research Center, "Stefan cel Mare" University, University Street, 13, Suceava 720229, Romania

Fax: +32-10472330. E-mail: s.radi@ump.ac.ma; yann.garcia@uclouvain.be

Contents

1. NMR ^{1}H and ^{13}C of ***L***……2
2. FT-IR spectra of Ligand and its coordination compounds……3
3. Powder XRD for coordination compounds……4-5
4. TGA data for ***L*** and **4**……6
5. HRMS……6-8
6. XRD analysis……9-13

NMR ^1H and ^{13}C of ligand *L*

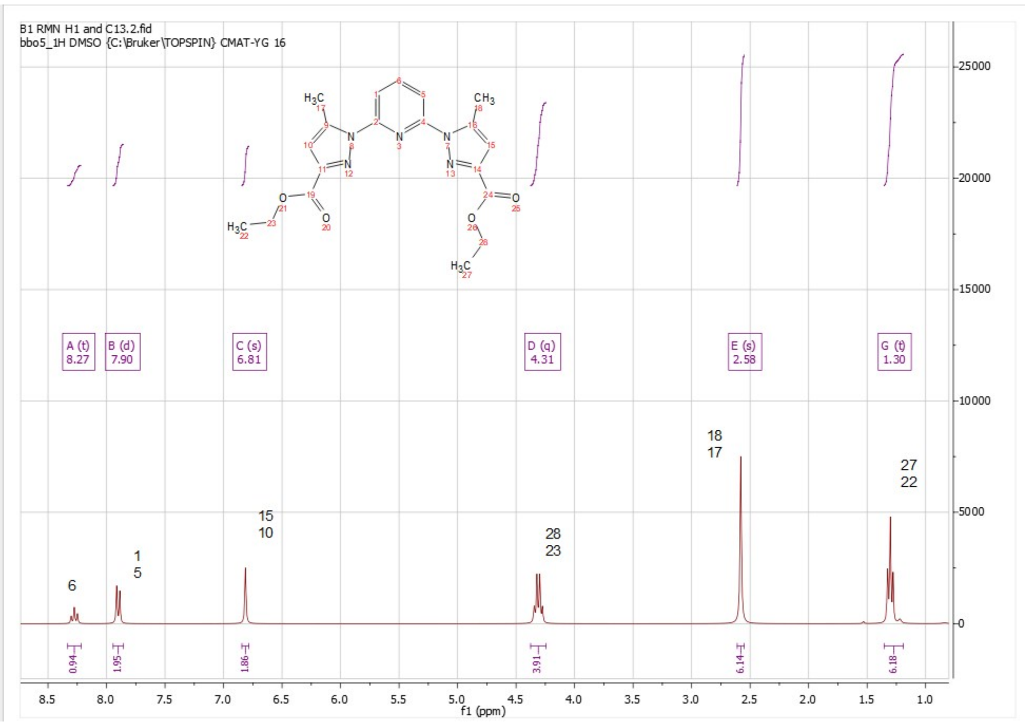

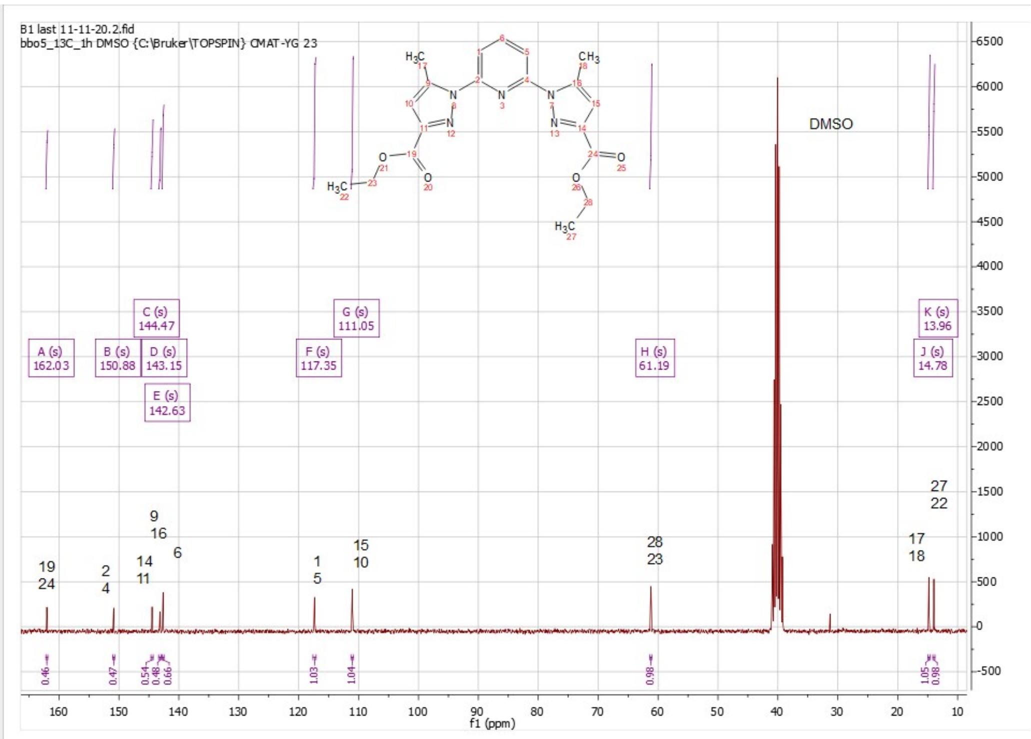

FT-IR spectroscopy comparison plot of *L* with 1-4.

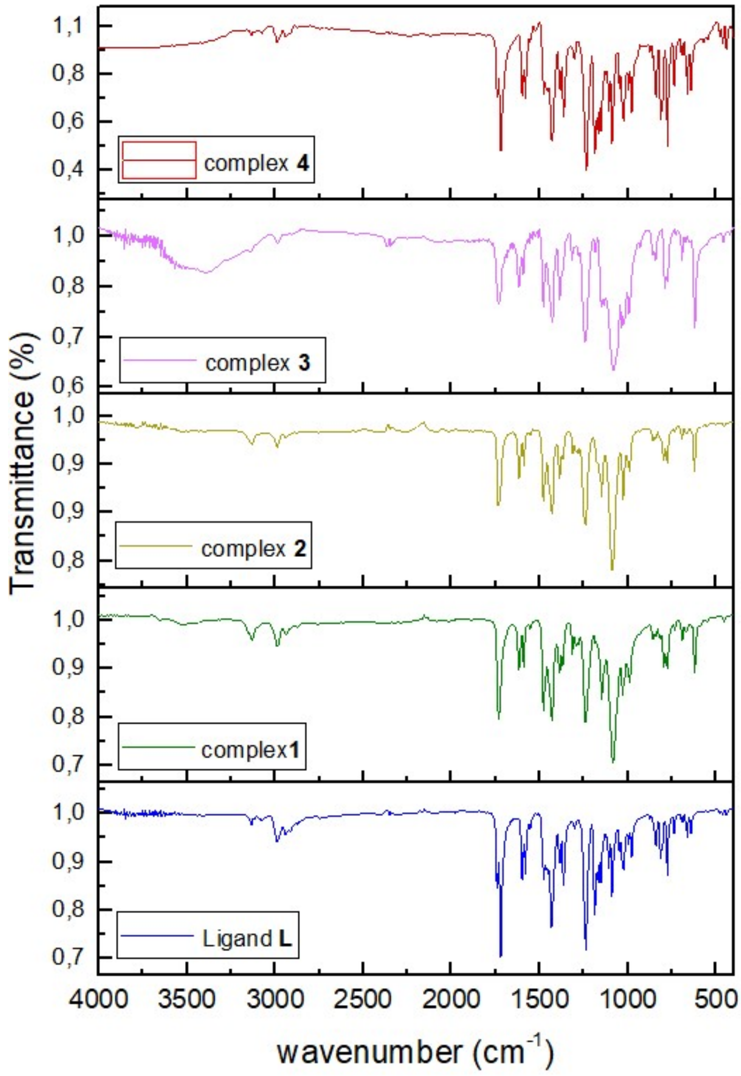

Powder X-ray diffraction of 1-4.

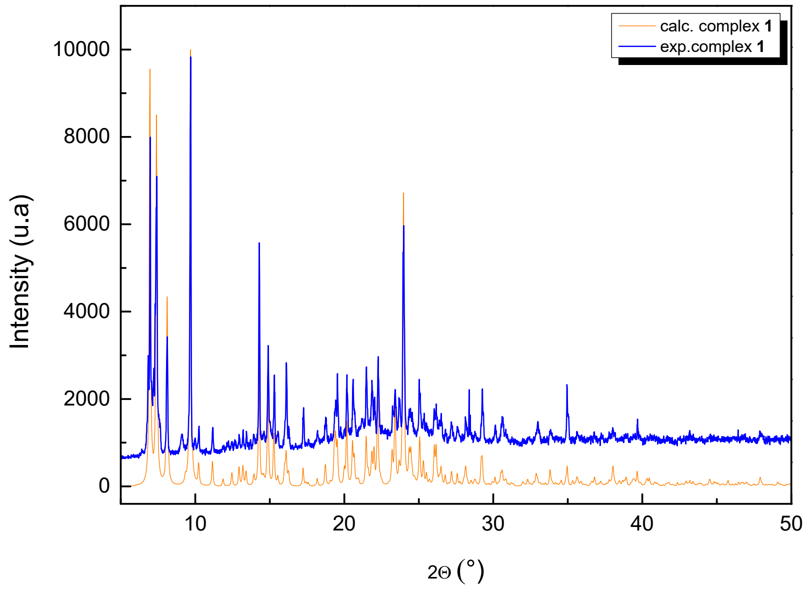

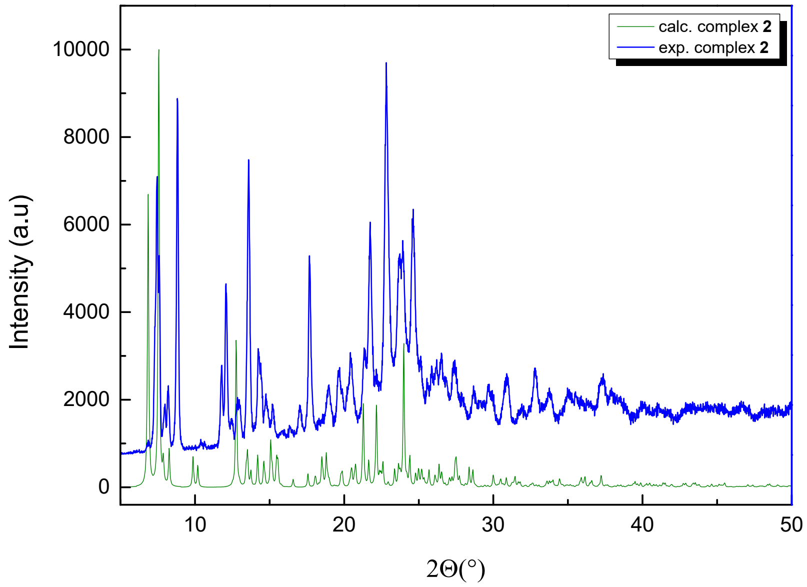

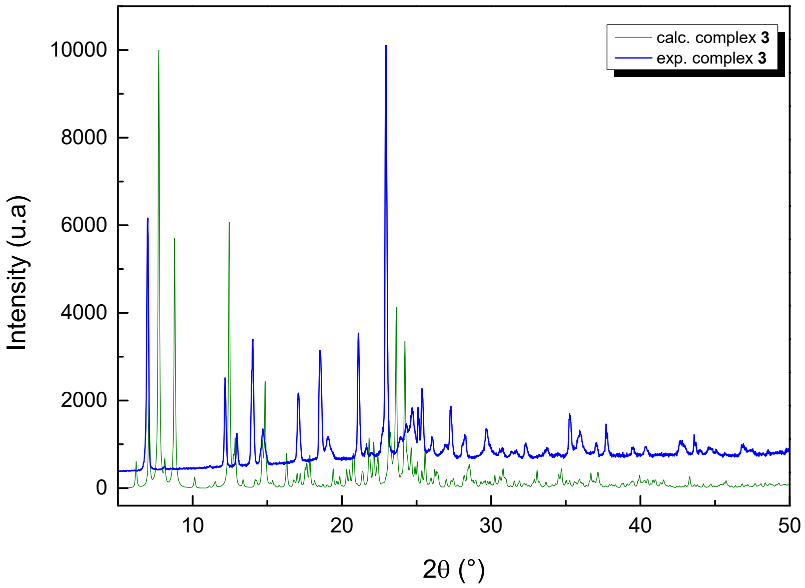
calc. complex 3
exp. complex 3
Intensity (u.a)
10000
8000
6000
4000
2000
0
10
20
30
40
50
2θ (°)

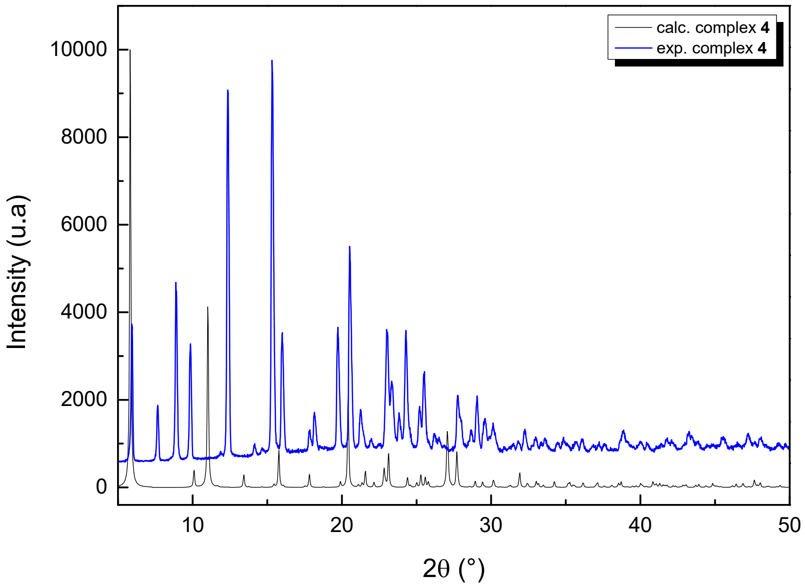
calc. complex 4
exp. complex 4
Intensity (u.a)
10000
8000
6000
4000
2000
0
10
20
30
40
50
2θ (°)

TGA of ligand *L* and 4.

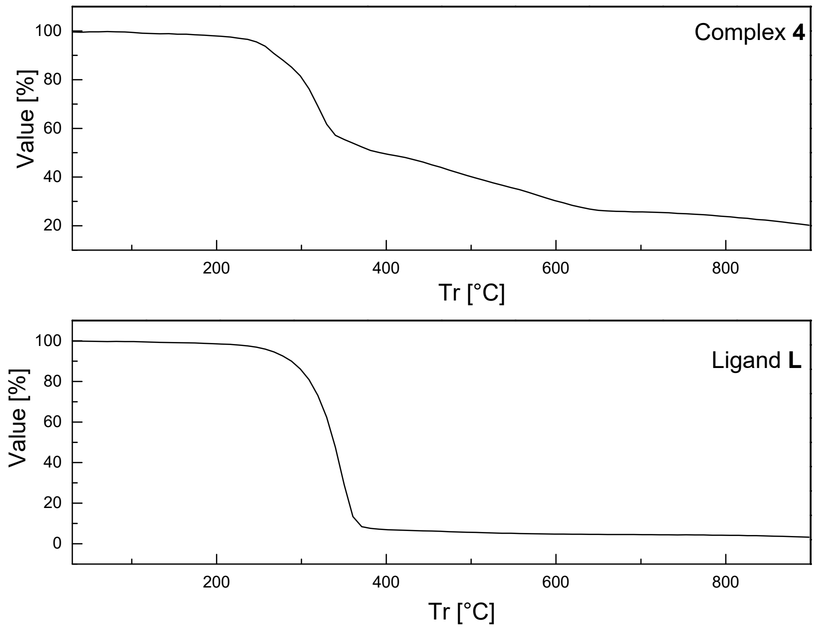

HRMS

Ligand ***L***

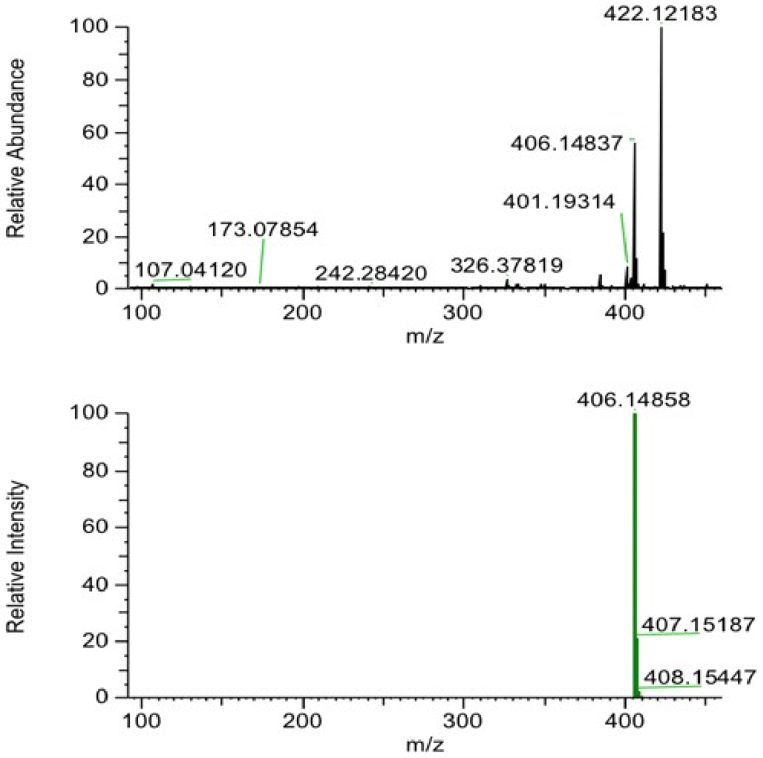

$[CoL_2](ClO_4)_2$ (**1**)

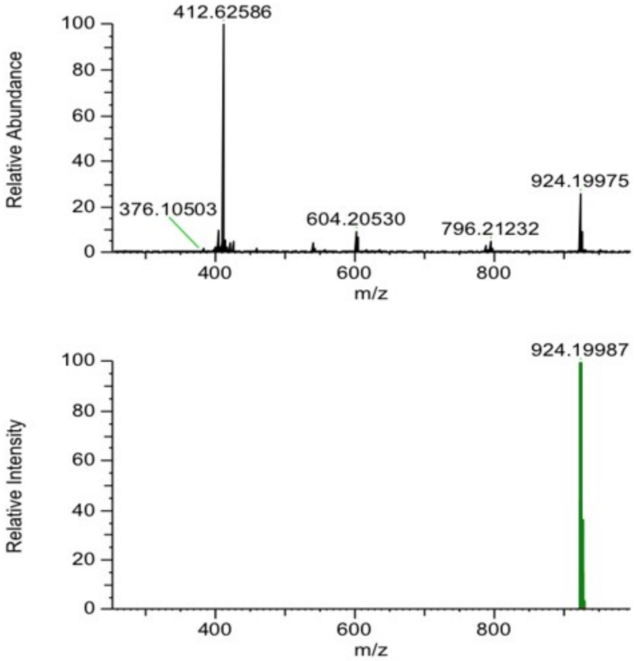

$[FeL_2](ClO_4)_2$ (**2**)

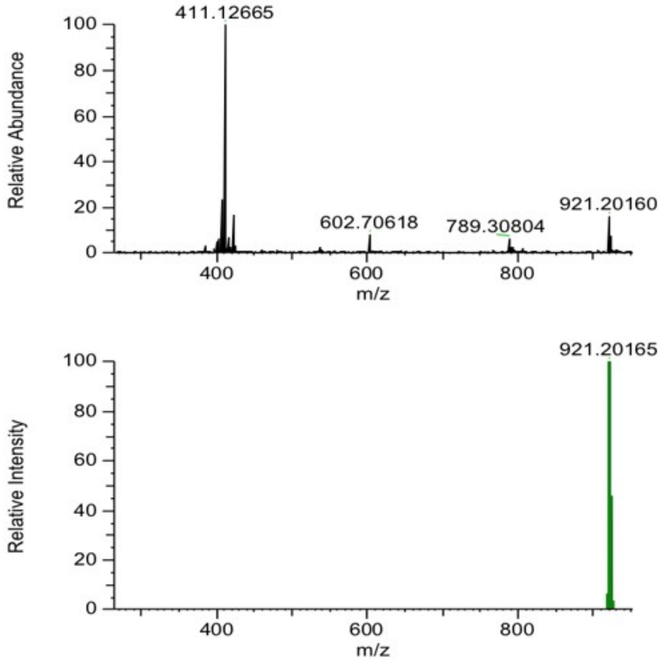

[NiL$_2$](ClO$_4$)$_2$ (**3**)

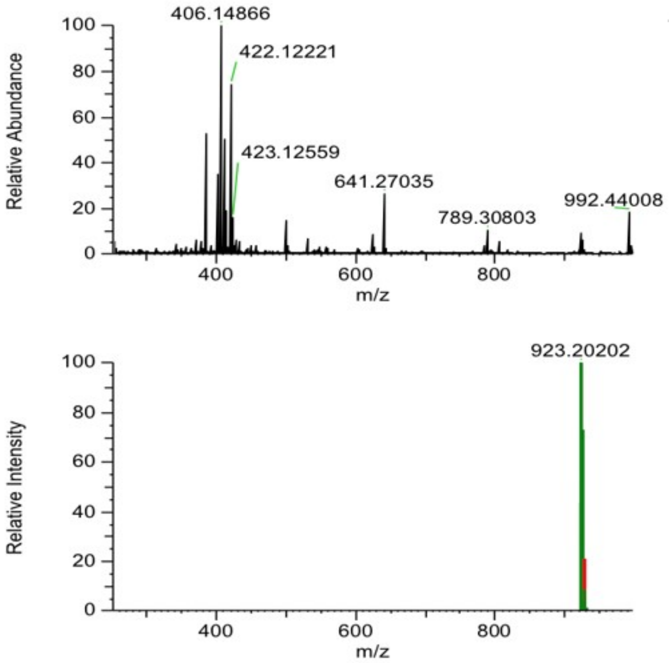

[MnLCl$_2$] (**4**)

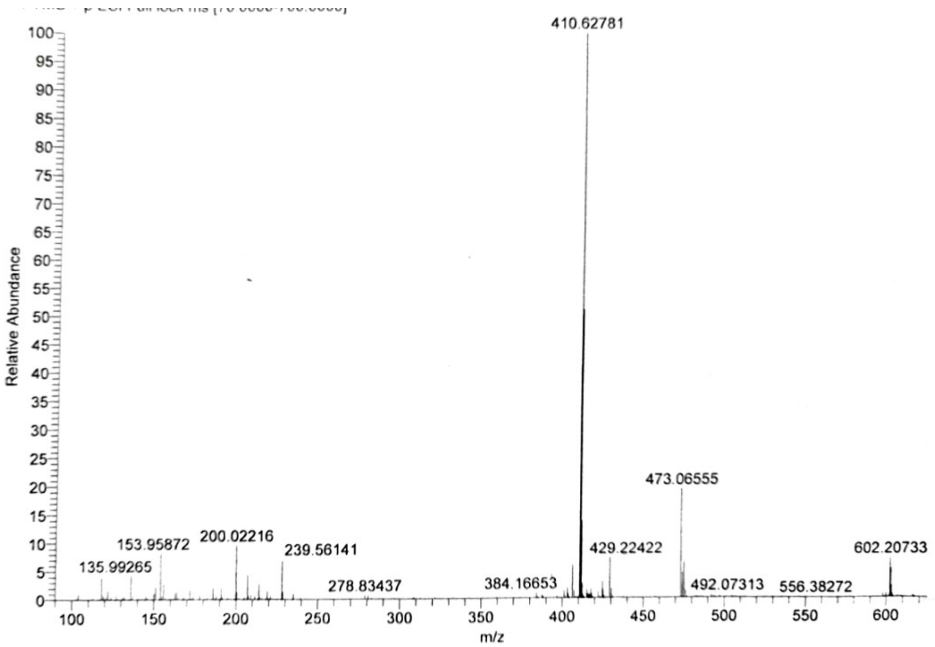

XRD analysis

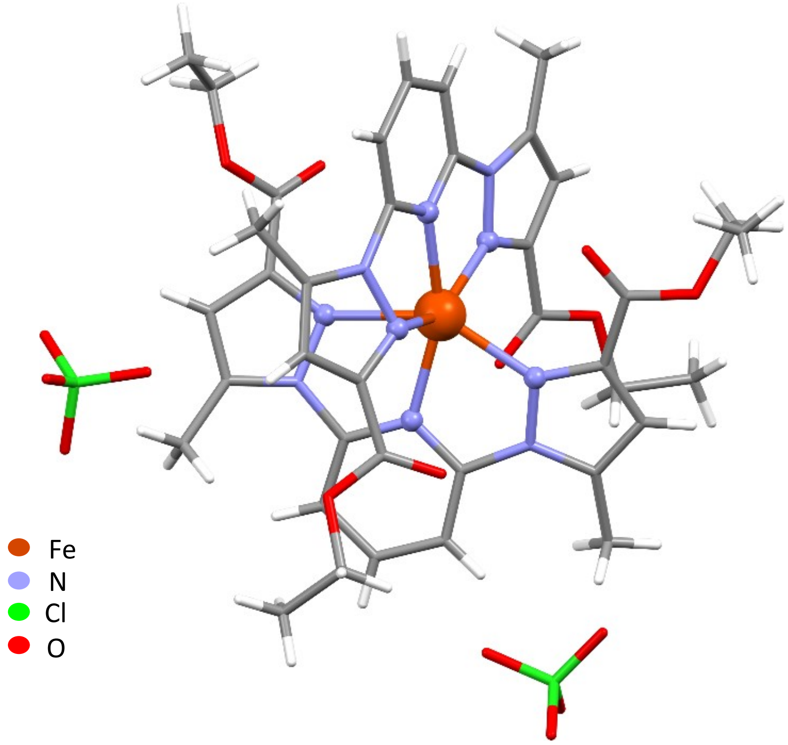

Figure S1: Perspective view of the molecular structure of **2**. Disorder was omitted for clarity.

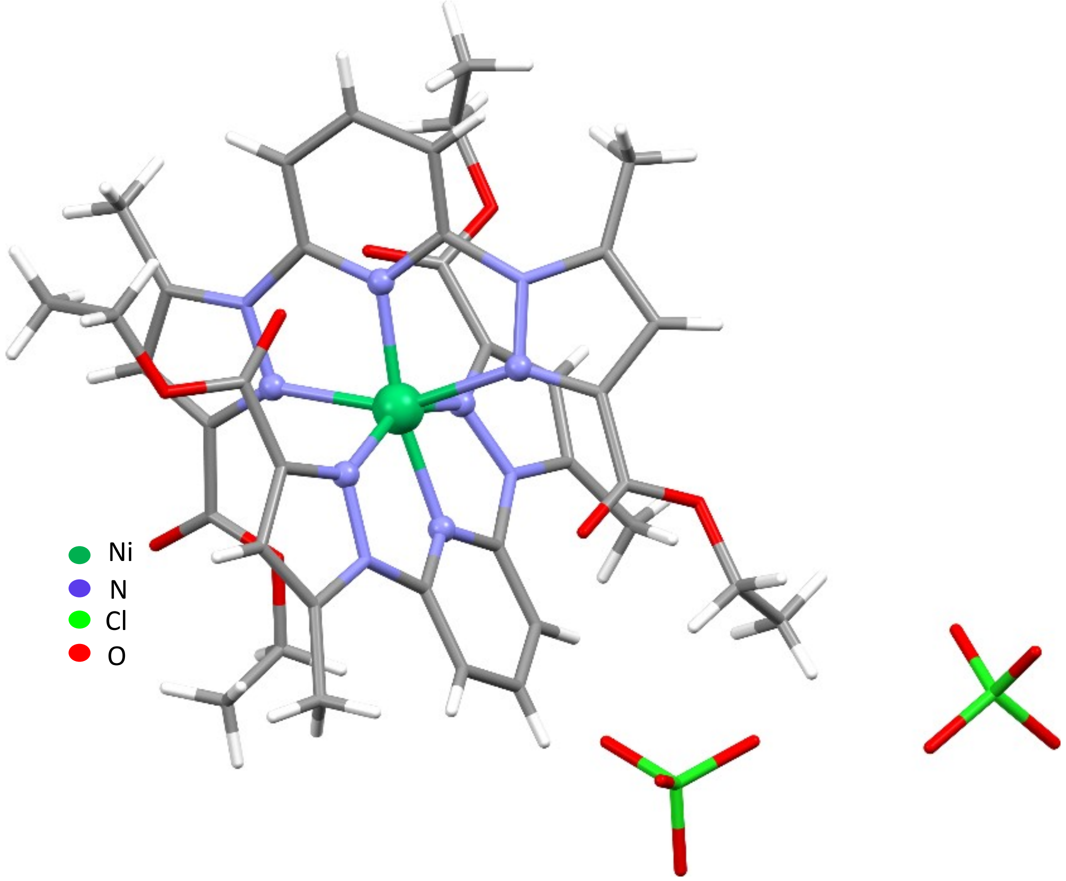

Figure S2: Perspective view of the molecular structure of **3**. Disorder was omitted for clarity.

1 **2** **3**

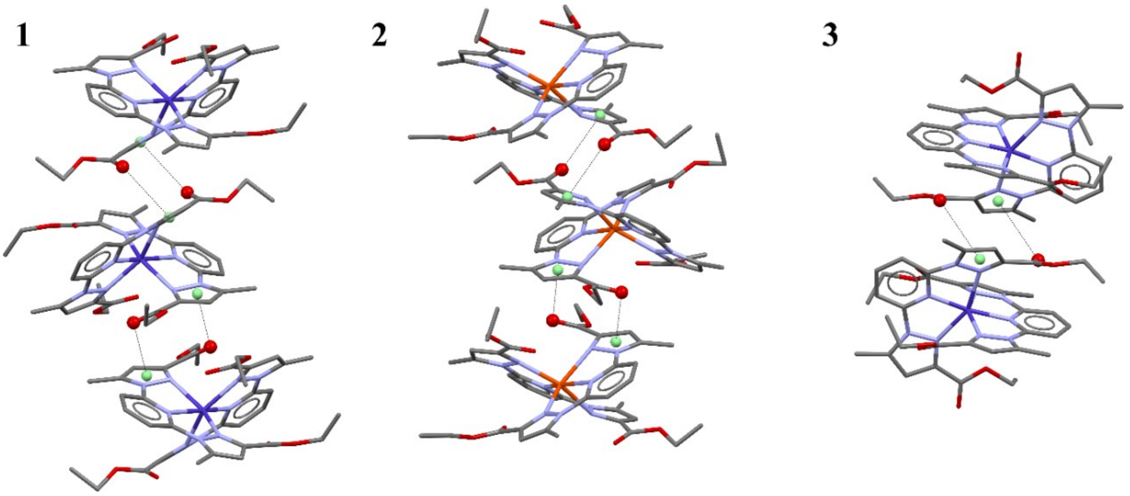

Figure S3: Packing motives found in complexes **1**, **2** and **3**, involving the twisted pyrazolyl ring centroids and the carbonyl oxygen.

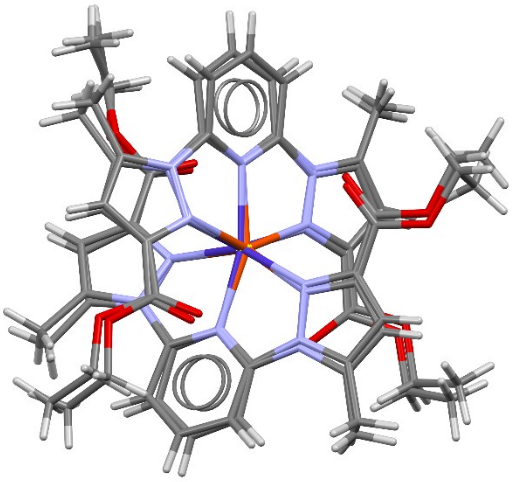

Figure S4: superposition of the Fe complex **2** on the Co complex **1**.

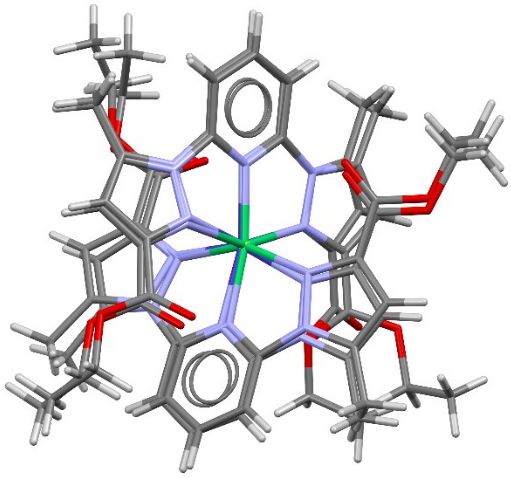

Figure S5: superposition of the Ni complex **3** on the Co complex **1**.

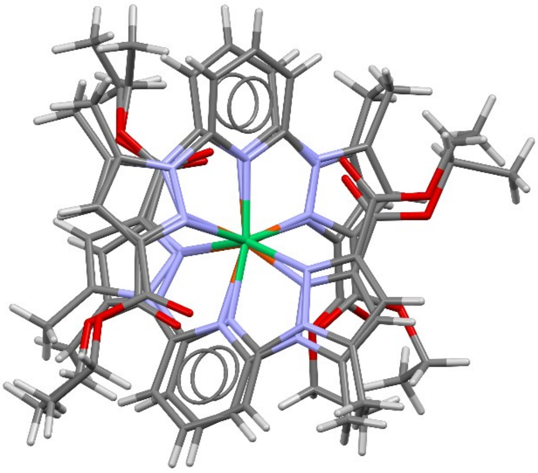

Figure S6: superposition of the Ni complex **3** on the Fe complex **2**.

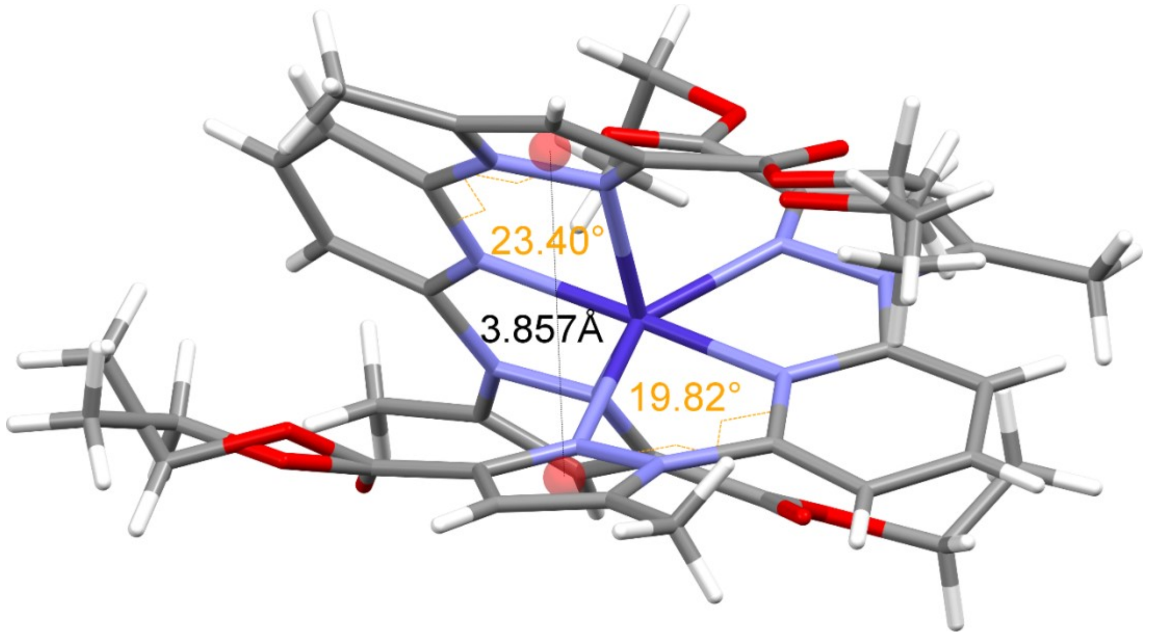

Figure S7: Torsion angles between the pyrazolyl and pyridyl rings, result in a short centroid-centroid distance between the pyrazolyl rings for **1**. Similar features are present in **2** and **3**.

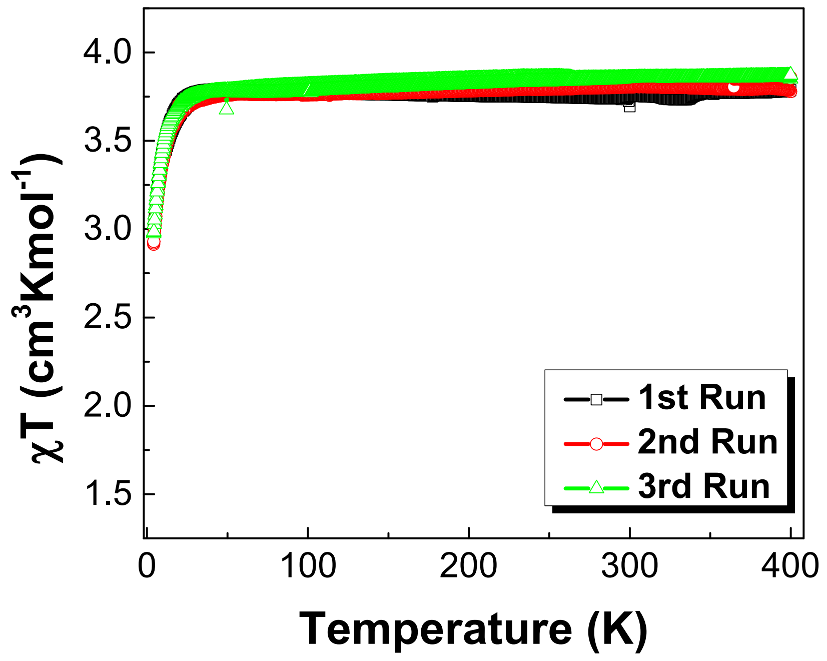

Figure S8 : Temperature-dependent $\chi_M T$ plot for **2** over three temperature runs, demonstrating the absence of any spin state crossover, over the whole temperature range investigated.

Table S1: Crystal data and structure refinements details for **1**-**4**.

Identification code	**1**	**2**	**3**	**4**
Empirical formula	$C_{38}H_{42}C_{l2}CoN_{10}O_{16}$	$C_{38}H_{42}C_{l2}FeN_{10}O_{16}$	$C_{38}H_{42}C_{l2}N_{10}NiO_{16}$	$C_{19}H_{21}Cl_2MnN_5O_4$
Formula weight	1024.64	1034.24	1024.42	509.25
T (K)	297(2)	297(2)	150(2)	297(2)
Wavelength (Å)	0.71073	0.71073	0.71073	0.71073
Crystal system	Monoclinic	Monoclinic	Monoclinic	Trigonal
Space group	$P2_1/n$	$P2_1/c$	$C2/c$	R-3c
Unit cell dim. *a* (Å)	12.8107(4)	14.3475(3)	29.5243(11)	30.3293(6)
b (Å)	14.1995(4)	25.7510(6)	13.8841(4)	30.3293(6)
c (Å)	25.5626(6)	13.6655(4)	22.5685(9)	13.6082(4)
β (°)	96.566(3)	114.200(3)	105.786(4)	
Volume (Å^3)	4619.5(2)	4605.2(2)	8902.4(6)	10840.7(5)
Z	4	4	8	18
Density (calc.) (g/cm^3)	1.473	1.492	1.529	1.404
Abs. coeff. (mm^{-1})	0.567	0.526	0.639	0.803
F(000)	2116	2137	4240	4698
Crystal size (mm^3)	0.50 x 0.30 x 0.10	0.47 x 0.30 x 0.08	0.50 x 0.40 x 0.20	0.50 x 0.30 x 0.25
θ range for data collection	3.201 to 25.700°.	2.794 to 25.243°.	2.846 to 25.689°.	2.686 to 25.690°.
Reflections collected	36203	30251	39648	30303
Independent reflections	8729 [$R_{(int)}$ = 0.0350]	8298 [$R_{(int)}$ = 0.0412]	8411 [$R_{(int)}$ = 0.0353]	2279 [$R_{(int)}$ = 0.0268]
Completeness to θ = 25.242°	99.4 %	99.5 %	99.4 %	99.2 %
Absorption correction	Semi-empirical from equivalents			
Max. and min. transm.	1.00000 and 0.74762	1.00000 and 0.37734	1.00000 and 0.71206	1.00000 and 0.83245
Refinement method	Full-matrix least-squares on F^2			
Data / restr. / param.	8729 / 136 / 762	8298 / 710 / 792	8411 / 10 / 628	2279 / 2 / 178
Goodness-of-fit on F^2	1.052	1.094	1.039	1.077
Final R indices [I>2sigma(I)]	R_1 = 0.0453, wR_2 = 0.1225	R_1 = 0.0652, wR_2 = 0.1625	R_1 = 0.0369, wR_2 = 0.0931	R_1 = 0.0313, wR_2 = 0.0830
R indices (all data)	R_1 = 0.0542, wR_2 = 0.1294	R_1 = 0.0881, wR_2 = 0.1780	R_1 = 0.0400, wR_2 = 0.0953	R_1 = 0.0334, wR_2 = 0.0844
$\Delta\rho$ max/min (e.Å^{-3})	0.611 and -0.239	1.151 and -0.266	0.515 and -0.452	0.203 and -0.179

Table S2: Bond lengths and bond angles around the metal centre for **1-3**.

Bond lengths	**1**	**2***	**3***
M-N_{ax} 1			
M-N_{ax} 2			
M-N_{eq} 1			
M-N_{eq} 2			
M-N_{eq} 3			
M-N_{eq} 4			
Bond angles			
$N_{Ax\,1}$-M-$N_{Ax\,2}$	165.39(8)	157.61(11)	168.21(6)
$N_{Ax\,1}$-M-$N_{eq\,1}$	75.09(7)	72.54(10)	77.29(6)
$N_{Ax\,1}$-M-$N_{eq\,2}$	94.60(7)	90.42(9)	97.96(6)
$N_{Ax\,1}$-M-$N_{eq\,3}$	74.44(7)	72.48(11)	76.19(6)
$N_{Ax\,1}$-M-$N_{eq\,4}$	116.90(7)	125.27(11)	109.03(6)
$N_{Ax\,2}$-M-$N_{eq\,1}$	94.37(7)	92.25(10)	91.74(6)
$N_{Ax\,2}$-M-$N_{eq\,2}$	75.12(7)	71.67(10)	76.99(6)
$N_{Ax\,2}$-M-$N_{eq\,3}$	117.28(7)	123.74(11)	114.78(6)
$N_{Ax\,2}$-M-$N_{eq\,4}$	74.29(7)	72.89(11)	76.51(6)
$N_{eq\,1}$-M- $N_{eq\,2}$	90.63(8)	85.50(10)	87.54(6)
$N_{eq\,1}$-M- $N_{eq\,3}$	148.04(7)	143.97(10)	153.46(6)
$N_{eq\,1}$-M- $N_{eq\,4}$	99.36(8)	101.08(11)	98.30(6)
$N_{eq\,2}$-M- $N_{eq\,3}$	101.18(8)	103.08(10)	97.05(6)
$N_{eq\,2}$-M- $N_{eq\,4}$	148.40(7)	144.17(10)	153.01(6)
$N_{eq\,3}$-M- $N_{eq\,4}$	86.01(8)	92.11(12)	89.41(6)

* Values for bond lengths and angles were obtained after superposition of **2** and **3** on **1** for better comparison between the complexes

Table S3. Bond lengths and bond angles for **4**.

Bond lengths	**4**
Mn $Cl2_{Ax}$	2.3359(5)
Mn $N3_{eq}$	2.3120(15)

Mn N9$_{eq}$	2.268(2
Mn N3$_{eq}$	2.3120(15)
Mn Cl2$_{eq}$	2.3359(5)
Bond angles	
Cl$_{Ax}$ Mn Cl$_{eq}$	133.83(4)
Cl$_{Ax}$ Mn N$_{eq1}$	105.20(4)
Cl$_{Ax}$ Mn N$_{eq2}$	113.2(5)
Cl$_{Ax}$ Mn N$_{eq3}$	90.94(4)
Cl$_{eq}$ Mn N$_{eq1}$	105.19(4)
Cl$_{eq}$ Mn N$_{eq2}$	112.9(5)
Cl$_{eq}$ Mn N$_{eq3}$	90.95(4)
N$_{eq1}$ Mn N$_{eq2}$ (*)	72.6(5)
N$_{eq1}$ Mn N$_{eq3}$	138.37(8)
N$_{eq2}$ Mn N$_{eq3}$ (*)	65.8(5)

(*) Neq2 is disordered around the 2-fold axis, hence the difference between both values

Table S4 RMSD and maximal displacement between complexes **1**, **2**, and **3**.

	RMSD (Å)	Max Deviation (Å)
Superposition of 2 on 1	0.5216	2.8204
Superposition of 3 on 1	0.7219	2.3544
Superposition of 3 on 2	0.8904	2.8630
